# Nature can suffer, too: behavioral evidence of empathy with ecosystems and its link to pro-environmental attitudes

**DOI:** 10.7717/peerj.21383

**Published:** 2026-06-26

**Authors:** Olivia Brunet, Axel Carlier, Maxime Cauchoix, Hélène Cochet, Elisabeth Fonteneau, Romain Guendon, Sébastien Roussel, Arielle Syssau, Gladys Barragan-Jason

**Affiliations:** 1 Station d’Ecologie Théorique et Expérimentale (SETE), CNRS, Moulis, France; 2 IRIT, Université de Toulouse, CNRS, Toulouse INP, France; 3 CLLE, Université de Toulouse, CNRS, Toulouse, France; 4 Department of Psychology, Univ. Montpellier Paul Valéry, Montpellier, France; 5 CEE-M, Univ Montpellier, CNRS, INRAE, Institut Agro, Montpellier, France

**Keywords:** Empathy with nature, Environmental attitudes, Human-nature interaction, Ecosystem care

## Abstract

**Background:**

As global environmental challenges intensify, understanding the psychological factors that foster pro-environmental actions is essential. This study examines whether empathy, traditionally studied in the context of human and animal relationships, can be extended to natural ecosystems, and how this broader empathy relates to pro-environmental attitudes.

**Methods:**

We analyzed self-reported empathic responses to distressing images of humans, animals, urban ecosystems, and natural ecosystems, from 122 participants. Participants also completed measures of trait empathy and pro-environmental attitudes.

**Results:**

Our findings show that empathy with humans is moderately to strongly correlated with empathy with animals and ecosystems, supporting the extension of empathic processes beyond human targets. Moreover, empathy with animals and natural ecosystems is significantly positively associated with pro-environmental attitudes

**Discussion:**

Overall, our findings provide behavioral evidence that empathy can extend to ecosystems and that empathy toward other-than-human beings may contribute to pro-environmental attitudes. We discuss implications for environmental psychology, as well as methodological considerations for future research and educational interventions.

## Introduction

Our planet is undergoing global environmental changes that are drastically affecting biodiversity and the climate (Intergovernmental Science-Policy Platform on Biodiversity and Ecosystem Services (IPBES), 2019; IPCC, 2023). Anthropogenic activities—such as intensive agriculture, increasing use of energy, waste production and urbanization—are acknowledged as the main drivers of these changes (Intergovernmental Science-Policy Platform on Biodiversity and Ecosystem Services (IPBES), 2019; IPCC, 2023). Despite various regulations and guidelines, achieving the United Nations’ Sustainable Development Goals remains challenging, particularly as climate change and biodiversity decline are rapidly increasing. Addressing these issues requires transforming individual and collective norms and values globally, at the social, political, environmental and economic levels (Intergovernmental Science-Policy Platform on Biodiversity and Ecosystem Services (IPBES), 2019).

A recent meta-analysis (Barragan-Jason et al., 2023) shows that improving psychological and physical connections between humans and other-than-human beings (Price & Chao, 2023) can serve as an important leverage point for achieving sustainability. In this context, sustainability is understood as a non-exploitative and care-oriented use of the environment (Richardson et al., 2020; Riechers et al., 2021). Importantly, enhancing such connections has been associated with an increase in pro-environmental behaviors and greater support for conservation policies. These positive effects have been observed both at the individual level (Barragan-Jason et al., 2023; Ives et al., 2017; Mackay & Schmitt, 2019; Soga & Gaston, 2024) and societal level, where social and institutional contexts shape patterns of connection and disconnection (Beery et al., 2023; Ives et al., 2018; Richardson et al., 2022; Richardson et al., 2025).

### Human-nature connectedness

The psychological connection with nature—commonly referred to as *human-nature connectedness*—has received increasing attention as a potential pathway toward a more sustainable future (*e.g.*, Barragan-Jason et al., 2022; Richardson et al., 2020). Higher levels of human-nature connectedness have been positively associated with enhanced physical, social and mental health. Moreover, it is linked to greater pro-environmental attitudes (*i.e.,* beliefs, values and feelings about environmental issues; Milfont & Duckitt, 2010; Moussaoui et al., 2016) as well as to behaviors (*e.g.*, recycling, supporting sustainable agricultural practices or using alternative means of transport; Barragan-Jason et al., 2022; Mayer et al., 2009; Nisbet, Zelenski & Murphy, 2009).

Human-nature connectedness has been primarily defined in environmental psychology as the extent to which individuals include other-than-human beings within their self (Schultz, 2002). This definition, which situates human-nature connectedness as a psychological construct including both affective and cognitive dimensions, has been the basis for most studies in environmental psychology (Barragan-Jason et al., 2022; Clayton, 2003; Mayer & Frantz, 2004; Schultz, 2002; Soga & Gaston, 2024). The cognitive dimension refers to beliefs and representations about the relationship between human and nature while the affective dimension includes emotional attachment and sense of belonging to the natural world (Nisbet, Zelenski & Murphy, 2009).

From this psychological standpoint, several self-report instruments have been developed to measure the construct. The Connectedness to Nature Scale (Mayer & Frantz, 2004) was designed to capture primarily the affective dimension of this construct whereas the Nature Relatedness scale (Nisbet, Zelenski & Murphy, 2009) includes emotional, cognitive, and experiential components. However, their length often limits their use in experimental contexts. Although shorter versions have been proposed (Nisbet & Zelenski, 2013; Pasca, Aragonés & Coello, 2017), they typically require adaptation and validation for non-English-speakers, and even the French validation of the Connectedness to Nature Scale involved item reduction to achieve satisfactory psychometric properties (Navarro, Olivos & Fleury-Bahi, 2017).

The most concise and reliable alternative is the Inclusion of Nature in Self (INS) scale (Schultz, 2002), a one-item visual measure of human-nature connectedness. The INS has been described as “accurate in capturing individual differences” while being much simpler to administer (Brügger, Kaiser & Roczen, 2011). The Illustrated version of the INS further improves accessibility and replicability, particularly among individuals with difficulties with abstraction (Kleespies et al., 2021).

**Figure 1:**
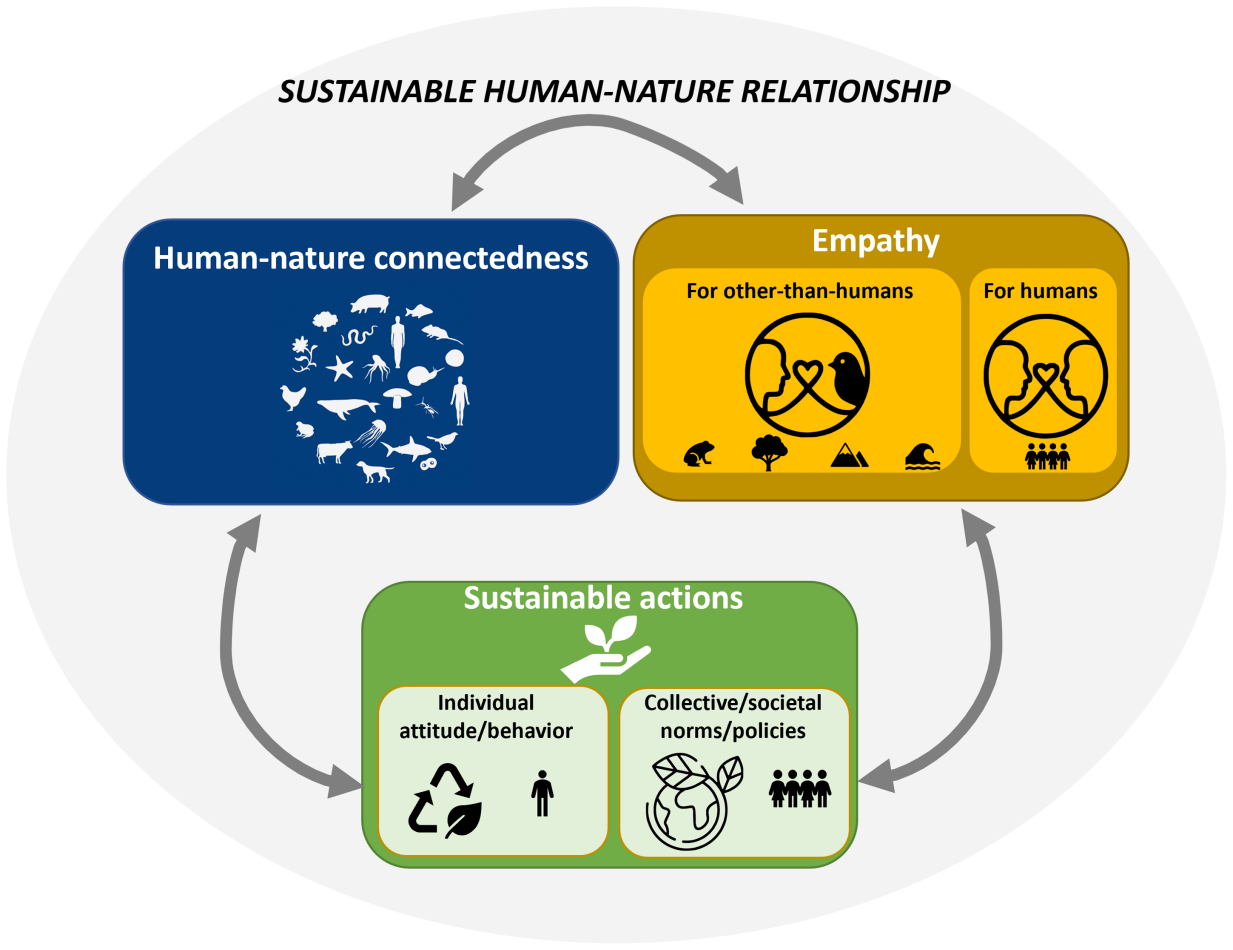
Schematic theoretical framework illustrating the relationships between human-nature connectedness, empathy and sustainable actions. Empathy is a broad construct encompassing including both humans and other-than-human beings (Research question 1). Empathy with other-than-human beings is linked to pro-environmental attitudes (Research question 2) as well as with human-nature connectedness (Research question 3). Previous studies already reported that high levels of human-nature connectedness are associated with high level of pro-environmental actions at individual (Barragan-Jason et al., 2023; Barragan-Jason et al., 2022) and societal levels (Richardson et al., 2020; Richardson et al., 2025). The framework supports the view that these three components are interconnected and represent key elements for fostering a sustainable future at individual, collective and societal levels.

Within the psychological framework of human-nature connectedness, empathy with other-than-human beings has been identified as a central emotional component, deriving from the concept of empathy with humans (Chawla, 2020; Cuff et al., 2016; De Vignemont & Singer, 2006; Decety & Lamm, 2006; Tam, 2013). In Chawla’s (2020) review on children’s connectedness with nature, empathy with other-than-human beings was argued to play a crucial role in fostering both affective connection with nature and pro-environmental behaviors. Thus, in the present study, we have conceptualized empathy as one of the key emotional mechanisms underpinning human-nature connectedness (Fig. 1).

### Empathy with humans

Empathy with humans is generally defined as the ability to both feel and understand another person’s emotional state while maintaining a clear self-other distinction (Cuff et al., 2016; Lepron, Causse & Farrer, 2015). It is commonly conceptualized as consisting of two dimensions: affective empathy and cognitive empathy (Vachon & Lynam, 2016). Affective empathy involves experience sharing, that is, a vicarious affective response, whether pleasant or unpleasant, congruent with the perceived target’s internal states (Wald et al., 2017). Cognitive empathy refers to perspective-taking, namely the ability to understand and imagine another’s situation, both at the individual level (putting oneself in another’s shoes) and at a broader socio-economic or political level that shapes others’ lived experiences (Segal, Wagaman & Gerdes, 2012). Together, these processes describe empathy as a psychological mechanism involving both emotional resonance and cognitive understanding.

In Batson’s framework, other-oriented feelings elicited by another’s distress—such as sympathetic, warm, or tender—are described as *empathic concern* and are distinguished from egoistically motivated reactions labeled as *personal distress* (Batson et al., 1991). While acknowledging this influential conceptualization, we adopt a more restrictive definition of empathy as the process of experience sharing and perspective-taking. We consider adjectives such as sympathetic, warm, or tender to reflect compassion, that is, an emotional response oriented toward the other. Because compassion may arise from empathic processes but can also stem from moral norms, religious beliefs, or self-interest (Tam, 2013; Wuthnow, 2012), we conceptually distinguish empathy, as a psychological process, from compassion, a related but broader emotional outcome.

Empirical research on induced empathy, also called state empathy, typically involves stimuli to trigger an empathic response. Although state empathy can be triggered by both positive and negative stimuli, most studies focus on negative emotional situations (*e.g.*, pain, distress) because these tend to evoke stronger and more immediate empathic responses, often associated with prosocial behaviors (Batson et al., 1991). State empathy can be experimentally induced through visual stimuli (*e.g.*, pictures or videos; Ibanez & Roussel, 2022) or stories (Li et al., 2024; Schultz, 2000). Perspective taking can be passive (simple exposure to the stimuli) or explicitly requested by the experimenter (Schultz, 2000). State empathy is mostly assessed using self-report questionnaires or electrophysiological measures (*e.g.*, skin electrodermal activity, electroencephalography, heart rate variability; Reniers et al., 2011; Vachon & Lynam, 2016).

Alternatively, trait empathy, rooted in personality psychology, is a general characteristic that predicts long-term patterns of attitudes and behaviors (Sevillano, Corraliza & Lorenzo, 2017) and is believed to grow over time (Kim & Cooke, 2021). It reflects a stable dispositional tendency, in contrast to state empathy, which captures momentary, context-dependent responses. Trait empathy can be assessed quantitatively through self-report measures, typically with validated questionnaires (Savard et al., 2022; Tam, 2013; Vachon & Lynam, 2016) which demonstrate good psychometric properties, including reliability and construct validity, across diverse populations.

### Empathy with nature

Empathy with nature, theorized about a decade ago, refers to the ability to share and understand the emotional experience of nature (Tam, 2013). Research on this form of empathy is relatively recent. Historically, psychology, economics, and conservation science have focused on the cognitive dimension of human-nature relationships, such as attitudes, values, and knowledge, rather than on emotional, affective, or relational processes and approaches to studying sustainability (Hoelle, Gould & Tauro, 2023; Pascual et al., 2017; Schultz, 2002; Tam, 2013).

As both trait and state empathy with humans foster prosocial behaviors (Batson et al., 1991), empathy with nature may likewise encourage pro-environmental attitudes (Ienna et al., 2022; Raymond et al., 2025; Wang et al., 2023). For instance, individuals with higher empathy with other-than-human beings, such as birds or trees, tend to exhibit more pro-environmental attitudes and a greater willingness to engage in environmental protection (Berenguer, 2007). To our knowledge, the Dispositional Empathy with Nature scale (Tam, 2013) is the only questionnaire designed to measure trait empathy with nature in distress.

Nature is a broad concept that varies strongly across knowledge systems. Here, we adopt the definition provided by Intergovernmental Science-Policy Platform on Biodiversity and Ecosystem Services (IPBES) (2019), which refers to the “non-human world, including coproduced features, with a particular emphasis on living organisms, their diversity, and their interactions among themselves and with their abiotic environment.” In other words, nature encompasses other-than-human living beings (animals, plants, fungi, bacteria, *etc.*), non-living components (rivers, rocks, mountains, *etc.*), and their interactions (Price & Chao, 2023). This broad conceptualization highlights the interdependence of biotic and abiotic elements in shaping the natural world.

Building on this, ecosystems, defined as a “dynamic community of living organisms (plants, animals and micro-organisms) in conjunction with the nonliving components of their environment, all interacting as a functional system” (Intergovernmental Science-Policy Platform on Biodiversity and Ecosystem Services (IPBES), 2019) are important components of nature. In this study, we adopt, and further use, the notion of “nature in distress” (Tam, 2013) to refer to situations in which ecosystems are significantly degraded or disrupted due to anthropogenic pressures. We posit that nature in distress encompasses biodiversity loss, habitat degradation, pollution, and alterations of key ecological processes that impair ecosystem functioning and resilience, consistent with established research on ecosystem degradation and environmental stress (Intergovernmental Science-Policy Platform on Biodiversity and Ecosystem Services (IPBES), 2019; Tam, 2013).

Despite the use of the word “nature” in the theorization of the concept of empathy with nature, most studies focus on empathic responses to animals or plants rather than on empathic response to entire ecosystems. Empirical studies on empathy with nature used pictures of other-than-human animals, such as whales or animals harmed by pollution (Schultz, 2000; Sevillano, Aragones & Schultz, 2007; Shelton & Rogers, 1981; Young, Khalil & Wharton, 2018). Other studies facilitate empathy using stories anthropomorphizing other-than-human animals (Li et al., 2024; Manfredo et al., 2020).

To date, only a limited number of studies have explicitly investigated empathy toward ecosystems as defined in the present framework. Geiger et al. (2017) found similar neural activation for suffering animals compared to degraded environments, suggesting similar neural patterns to empathic responses of humans, animals and environment. However, the activation was weaker for the environment, suggesting that participants may consider ecosystems to be experiencing entities capable of feelings but to a lesser extent than they do for human or animals. In another study, the authors examined whether visitors of a park were able to “think like a park” by asking them through a survey to take a park’s perspective (Walker & Chapman, 2003). They demonstrated that the participants could take the park’s perspective and that the extent of their empathic responses, was related to pro-environmental intentions. Another study conducted by Zhou & Wang (2022) has shown a positive relationship between negative anthropomorphizing of the planet and pro-environmental behavior.

Our current study therefore measured trait empathy with humans and with other-than-human beings, as well as state empathy induced by pictures of humans, other-than-human animals, urban and natural ecosystems in distress (represented by landscape photographs), in a sample of 122 participants. Indeed, given that a landscape is defined as “an area of land that contains a mosaic of ecosystems, including human-dominated ecosystems” (Intergovernmental Science-Policy Platform on Biodiversity and Ecosystem Services (IPBES), 2019), images of landscape can be regarded as an ideal proxy for the visual perception of dynamic ecosystems (Geiger et al., 2017). As a first step, we focused on empathy with ecosystems in distress since most existing studies on empathy have examined negative forms (Tam, 2013). We used a “passive viewing” procedure to investigate participants’ spontaneous self-reported empathic response.

This behavioral study allowed us to address three main research questions (RQ; Fig. 1): (RQ1) To what extent does the concept of state empathy with ecosystems relates to state empathy with humans and other-than-human animals? (RQ2) Is there a positive link between state empathy with ecosystems and pro-environmental attitudes? (RQ3) Is there a relationship between state empathy with ecosystems and human-nature connectedness? In doing so, the current study fills critical research gaps in expanding the concept of empathy with humans to empathy with ecosystems and explores how this might be linked to pro-environmental attitudes.

## Materials & Methods

### Participants

A power analysis was performed using the G*Power 3.1.9.7 statistical software to determine the sample size. For an expected medium effect size (*r* = 0.3; Cohen, 1988), a significant level (*α*) of 0.05 and a desired power (1 − *β*) of 0.95, the sample size required was 115 participants. One hundred and twenty-four French participants were recruited on the Université Paul Valéry—Montpellier III campus (Montpellier, France). Two participants were excluded from the analyses due to incomplete participation in the study.

In total, 122 participants (40 men, 81 women and one “other”; M_age_ = 21.57 years old; SD = 4.82) took part in the study at the platform PEACH (Platform for the Study and Analysis of Human Behavior). Most of the participants were students in Bachelor degree (78.7%). The distribution of their origin was relatively uniform (Rural: 24.6%; Semi-rural: 30.3%, Urban: 26.2%; City-center: 18.9%). Detailed descriptive statistics are available in Supporting Information (Table S1). Participants signed informed consent and the protocol was approved by the local ethics committee (reference number IRB00013686- 2023-04-CER UPVM—Université Paul Valéry—Montpellier III (France) IRB #2).

### Stimuli

#### Stimuli selection

Forty pictures (all resized to 981 × 720 pixels) were extracted from three affective standardized image databases: Open Affective Standardized Image Set (Kurdi, Lozano & Banaji, 2017), French Affective Images of Climate Change (Ottavi, Roussel & Syssau, 2021) and Geneva Affective Picture Database (Dan-Glauser & Scherer, 2011). These databases contain royalty-free images^1^ 1Except the Geneva Affective Picture Database, for which the information could not be found in the manuscript.that have been normatively evaluated along affective dimensions by hundreds of participants^2^ 2Open Affective Standardized Image Set (*N* = 822); French Affective Images of Climate Change (*N* = 106); Geneva Affective Picture Database (*N* = 60).using standardized rating scales, providing reliable normative mean scores for selecting emotional stimuli. We selected images that were available in the databases without considering ecosystem types, species diversity, or other criteria such as familiarity with the ecosystems. Our main inclusion criteria were to ensure the images elicit the appropriate valence (*i.e.,* the degree of negative affective response) and that the arousal (*i.e.,* the intensity of the affective response) and valence values were uniform across categories. Pictures can be provided upon request to the platform PEACH or the ethics committee of the Paul Valéry University.

#### Stimuli description

The arousal and valence values from the databases used different numeric rating scales, which were rescaled to a 0–100-point scale, with 0 representing a negative valence and 100 a positive valence. Twenty pictures with negative valence (*M* = 13.24; SD = 17.10) and twenty pictures with positive valence (*M* = 79.93; SD = 15.41) were selected to create four categories, each consisting of five positive and five negative valence images: human (*e.g.*, an injured adult, an adult sleeping in the street, a child starving, a happy child eating, people having fun at a party), animal (*e.g.*, an injured dog, a caged pig, a happy dog, a free wolf), natural ecosystem (*e.g.*, a clear-cut forest, a plastic polluted soil, a colorful mountain landscape, a lake), and urban ecosystem (*e.g.*, a flooded city, a junkyard, a sidewalk in a village, modern skyscrapers). Positive-valence pictures were also included in our design to counterbalance the effects of the negative stimuli and soften the unpleasant experiences of participants over time. Means of valence (V) and arousal (A) reported in our sample (Table S2; Figs. S1–S2) were significantly different for each category, except between humans and animals (V: *t* (241.2) = 0.94, *p* = .35; A: *t* (237.3) = −0.8, *p* = .40) and natural and urban ecosystems (V: *t* (241.5) = −0.7, *p* = .49; A: *t* (238.0) = 1.34, *p* = .18).

### Procedure

**Figure 2:**
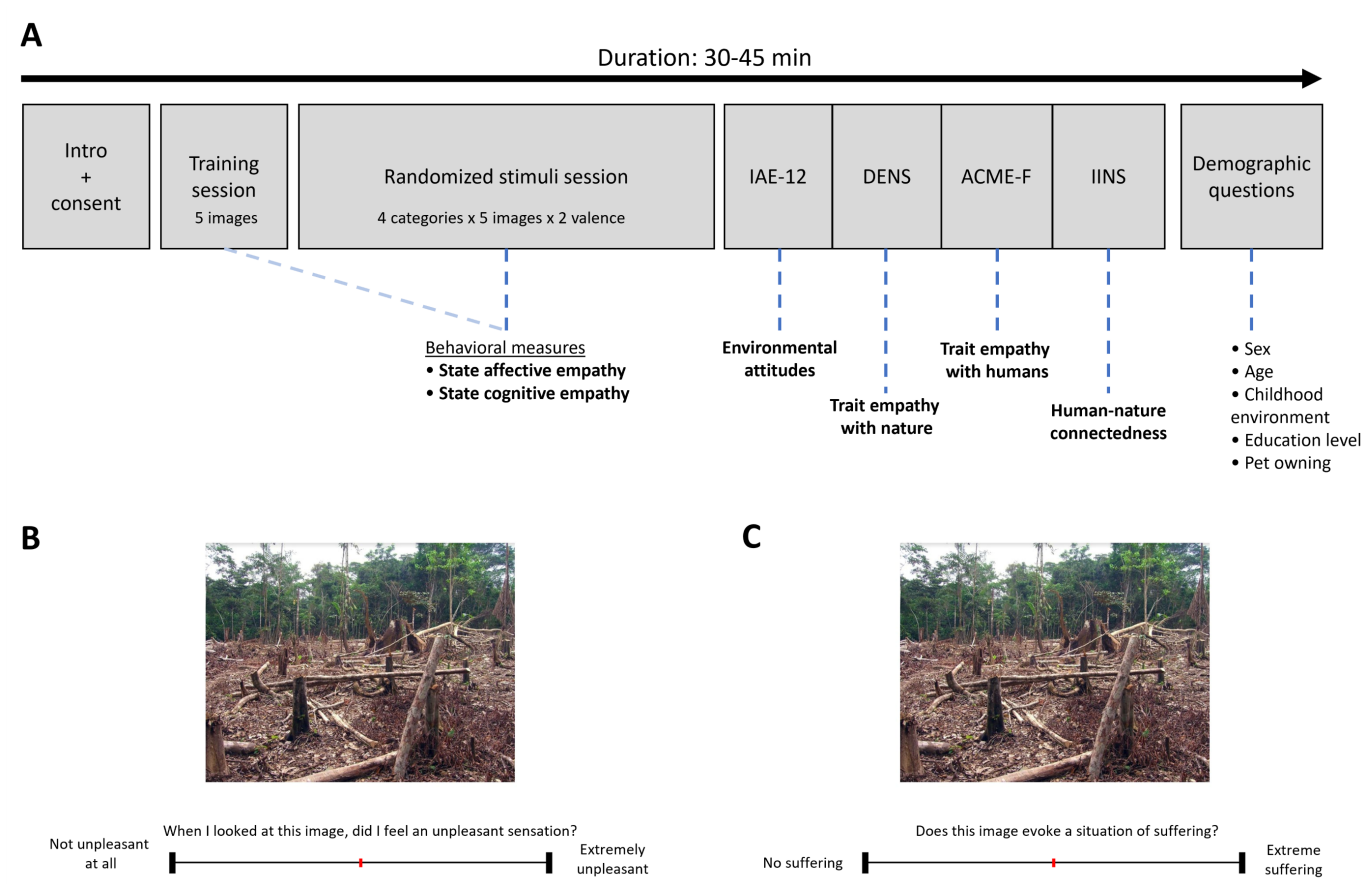
Flowchart of the study’s procedure and stimuli presentation. (A) After an introduction (Intro) explaining the task, participants gave their informed consent. They then performed a training session to verify their understanding of the instructions, followed by the actual task and the completion of a series of questionnaires. IAE-12 corresponds to the Environmental Attitudes Inventory, DENS is the Dispositional Empathy with Nature Scale, ACME-F corresponds to the Affective and Cognitive Measure of Empathy and IINS to the Illustrated Extension of the Inclusion in Self Scale. For each image, the questions appeared in the following order: (B) affective empathy; (C) cognitive empathy. There was no time limitation to answer. The following question appeared directly after the participant gave the answer to the preceding question.

The study, designed with the E-Prime 3.0 software, lasted around thirty to forty-five minutes and was divided in five main parts: (a) introduction and informed consent; (b) training session; (c) behavioral session; (d) questionnaires; (e) demographic questions (Fig. 2A). Upon arrival at the laboratory, participants received a general introduction to the study. The research was presented as a study on emotional reactions to visual content, and participants were informed of their right to withdraw at any time.

After signing the informed consent, participants first completed a series of five training images, followed by the actual stimuli (Fig. 2A). The order of stimuli presentation was randomized. Each picture was displayed on the screen until the participant answered four questions, using sliding scales ranging from 0 to 100. The sliding scales measured the participants’ emotional (valence and arousal) and empathic (affective and cognitive) responses. The two variables of empathy were measured in the following order: state affective empathy (Fig. 2B) and state cognitive empathy (Fig. 2C).

State affective empathy focuses on the degree of unpleasantness felt by participants while state cognitive empathy focuses on the degree of suffering evoked by the target in the image (human, animal or ecosystem). We used a general sentence to avoid influencing participants’ interpretation of the empathy target, given that they may focus either on the ecosystem as a whole or on its individual components (*e.g.*, trees in a forest), and may not attribute suffering to an ecosystem per se. For this reason, we deliberately avoided using the term “ecosystem” in the general formulation. Specifically, state affective empathy was measured using the following question: “*En regardant cette image, ai-je ressenti une sensation désagréable?*” (When I looked at this image, did I feel an unpleasant sensation?). Answers ranged from 0 –“*Pas du tout désagréable*” (Not unpleasant at all) to 100 –“*Extrêmement désagréable*” (Extremely unpleasant). State cognitive empathy was measured using the following question: “*Cette image évoque-t-elle une situation de souffrance?*” (Does this image evoke a situation of suffering?). Answers ranged from 0 –*“Aucune souffrance”* (No suffering) to 100 –*“Souffrance extrême”* (Extreme suffering).

The wording of the sentences was based on Lepron, Causse & Farrer (2015) and adapted after extensive discussions among the authors. We conducted a pre-test with 20 individuals from the target population that confirmed that all sentences were correctly understood. No substantial modifications were required following the pre-test.

### Questionnaires

Overall, participants completed four self-report questionnaires, which measured trait empathy with humans, trait empathy with other-than-human beings, pro-environmental attitudes and human-nature connectedness (Tables S3–S5; Fig. S3). In a preliminary analysis, an additional questionnaire was administered to evaluate the validity of our stimuli (*i.e.,* to explore whether the valence and arousal of our sample were comparable to the published databases) and of our measures (*e.g.*, potential impact of social desirability; see Table S6). After completing questionnaires, participants provided demographic information, including sex, age, childhood living environment, level of education and whether they had a pet during their lifetime. The authors have permission to use those instruments from the copyright holders.

#### ACME-F—Trait empathy with humans

The Affective and Cognitive Measure of Empathy (ACME; Vachon & Lynam, 2016) is a reliable measure of affective and cognitive trait empathy composed of thirty-six items (Table S3). The French translated and validated version (ACME-F; Savard et al., 2022) was used in our study (*α* = 0.85, *ω* = 0.88). The score was calculated by summing the scores for each item, accounting for reverse-scored items. Since twenty-four items assessed the affective dimension of trait empathy with humans and twelve items assessed the cognitive dimension, sub-scores for trait affective empathy and trait cognitive empathy were calculated in order to perform exploratory analyses.

#### DENS—Trait empathy with nature

Tam (2013) developed the seven-point Dispositional Empathy with Nature (DEN) scale, aimed to measure trait empathy with nature in distress, and more specifically plants and animals. This scale is composed of ten items (Table S4) measuring empathy with nature as a unidimensional concept. On our sample of participants, internal consistency was high (*α* = 0.89). The total score was calculated by summing the score obtained for each item.

#### IAE—Pro-environmental attitudes

The IAE-12 (Moussaoui et al., 2016) is the French translated and shortened version of the Environmental Attitudes Inventory (Milfont & Duckitt, 2010). This version is a twelve items self-report questionnaire (Table S5) aimed to measure pro-environmental attitudes in a two-dimensional manner: preservation and utilization. Preservation represents the belief that the priority must be to preserve nature and species diversity in their original state; while utilization represents the belief that it is normal and appropriate for the human species to use and modify nature and other species for personal reasons. The French translation of the shortened version respects the psychometric properties of the original questionnaire. However, on our participants’ sample, the internal consistency was low (*α* = 0.48, *ω* = 0.54), even on the preservation (*α* = 0.35) and utilization factors (*α* = 0.27) separately. The score was calculated by summing the score obtained for each item, taking into account reverse scored items.

#### IINS—Human-nature connectedness

The Illustrated Extension of the Inclusion of Nature in Self Scale (IINS; Kleespies et al., 2021; Schultz, 2002) is a one item questionnaire aimed to measure human-nature connectedness (Fig. S3). It has been chosen for its rapidity to fill in and understandability for French-speakers. Scores ranged between 1, meaning low connectedness with nature and 7, meaning high connectedness with nature, with higher scores indicating a greater perceived overlap between self and nature.

#### Social desirability

The social desirability questionnaire (Table S6) was assessed using the Marlow-Crowne Social Desirability Scale (Crowne & Marlowe, 1960; Sârbescu, Costea & Rusu, 2012) in order to control for participants’ tendency to respond in a way that would please the experimenter. Except trait cognitive empathy with humans (Spearman’s rho: −0.26, *p* = .005) and trait affective empathy with humans (Spearman’s rho: −0.39, *p* < .001) which negatively correlate with social desirability, no other significant correlation was found between social desirability scores and other measures. As social desirability bias is generally reflected in positive associations with socially valued traits, the absence of positive correlations suggests that socially desirable responding did not systematically inflate the measures.

### Data analysis

All analyses were performed using the R programming language on the RStudio/2024.04.2+764 interface. We performed a Kolmogorov–Smirnov test to assess the normality of our data. Because the test of normality was not significant for some key variables, we conducted a series of Spearman’s correlations to investigate (1) the relationships between empathic responses across image categories (research question 1), (2) the link between empathic responses and pro-environmental attitudes (research question 2) and (3) the link between empathic responses and human-nature connectedness (research question 3). To correct for multiples comparisons, we applied the Benjamini–Hochberg correction based on the number of correlations calculated for each research question.

In order to investigate the link between the potential effects of socio-demographic information on empathic responses, we conducted an exploratory analysis including eight linear regressions. We normalized the following variables: state affective empathy with humans, and state affective and cognitive empathy with animals (Table S7). Exploratory analyses were also conducted to examine empathy ratings across image categories (human, animal, natural ecosystems, urban ecosystems) and types of empathy (cognitive, affective). A 2 (empathy type: cognitive, affective) ×4 (image category) repeated-measures ANOVA was performed. *Post-hoc* pairwise comparisons were conducted using the Benjamini–Hochberg procedure to control the false discovery rate (Table S8).

## Results

### Relations between empathy with humans and empathy with other-than-human beings

#### Correlations between state empathy measures

**Figure 3:**
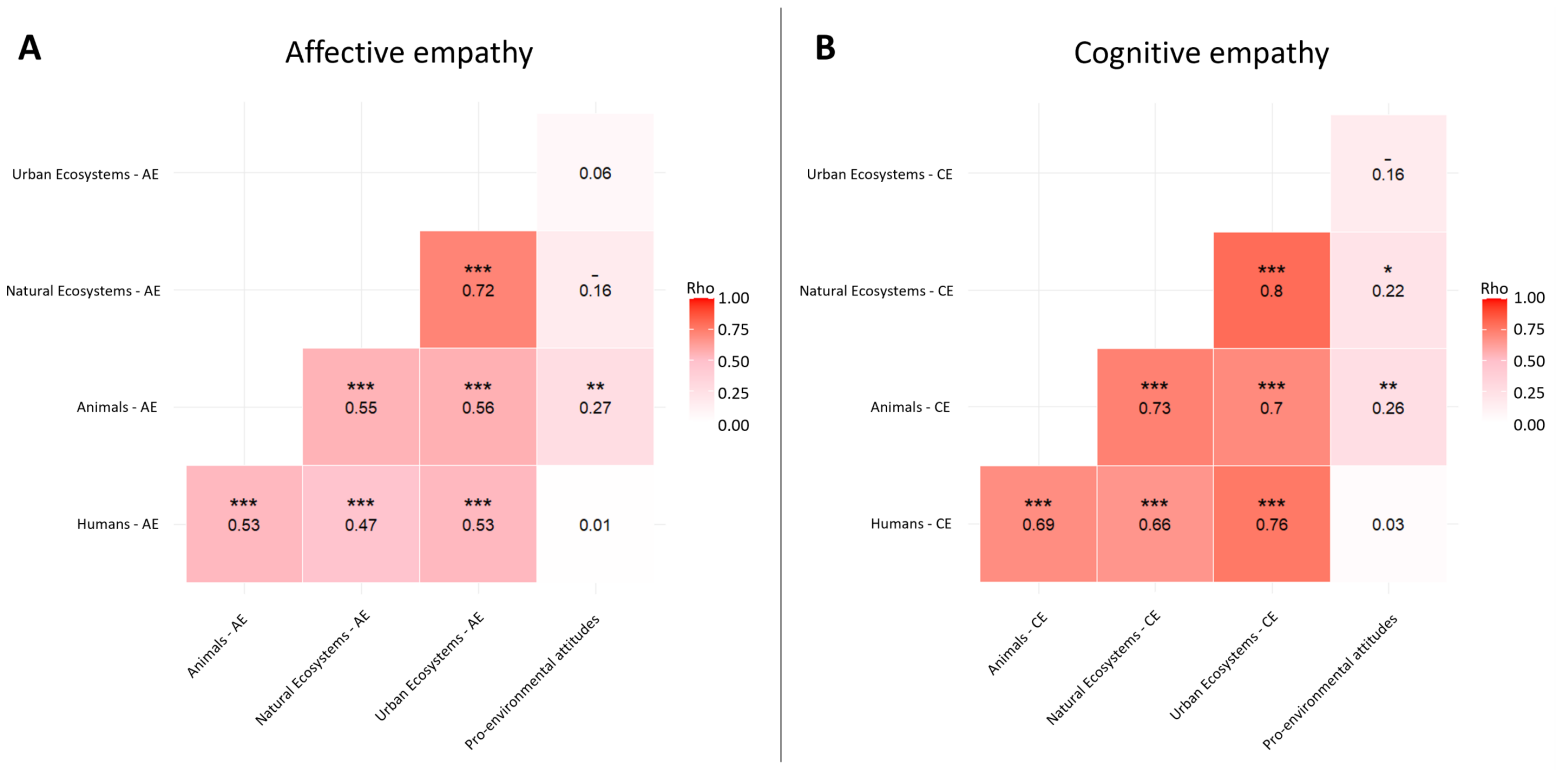
Correlation matrices between categories of state affective empathy (AE) in (A) and state cognitive empathy (CE) in (B). Natural Ecosystems (AE and CE), Animals (AE and CE), Humans (AE and CE) and Urban Ecosystems (AE and CE) correspond to the four categories of pictures, depending on the measured dimension of empathy (affective or cognitive). Red squares represent a positive and significant correlation, and contain the value of the associated Spearman’s rho coefficient. The closer the coefficient is to 1, the stronger is the positive association between two variables. * *p*-values < .05; ** *p*-values < .005; *** *p*-values < .001; -: *p*-values < 0.10.

We found moderate-to-strong, significant and positive correlations between state empathy with humans and state empathy with animals, natural ecosystems, and urban ecosystems, for both affective empathy (Fig. 3A) and cognitive empathy (Fig. 3B; Table S9). Specifically, empathy with humans was strongly, significantly and positively correlated with empathy with animals (Affective Empathy: Spearman’s rho = 0.53, *p* < .001; Cognitive Empathy: Spearman’s rho = 0.69, *p* < .001), empathy with natural ecosystems (Affective Empathy: Spearman’s rho = 0.47, *p* < .001; Cognitive Empathy: Spearman’s rho = 0.66, *p* < .001), and empathy with urban ecosystems (Affective Empathy: Spearman’s rho = 0.53, *p* < .001; Cognitive Empathy: Spearman’s rho = 0.76, *p* < .001). All correlations remained significant after correction for multiple comparisons.

#### Correlations between state empathy measures and trait empathy with other-than-human beings

We found moderate, positive and significant correlations between trait empathy with other-than-human beings and all state empathy measures (Table S9; Fig. S4). The strongest correlations were found between trait empathy with other-than-human beings and (a) affective empathy with animals (Spearman’s rho = 0.38, *p* < .001; Fig. S4A), (b) cognitive empathy with animals (Spearman’s rho = 0.289, *p* = .001; Fig. S4D) and (c) cognitive empathy with natural ecosystems (Spearman’s rho = 0.32, *p* < .001; Fig. S4E). All correlations remained significant after correction for multiple comparisons.

#### Correlations between trait empathy measures

We found positive moderate correlations between trait empathy measures. Trait empathy with other-than-human beings was positively correlated with trait empathy with humans (Spearman’s rho = 0.34, *p* < .001; Table S9). All values retained the same significance after correction for multiple comparisons.

### Relations between empathy with other-than-human beings and pro-environmental attitudes

#### Correlation between state empathy and pro-environmental attitudes

**Figure 4:**
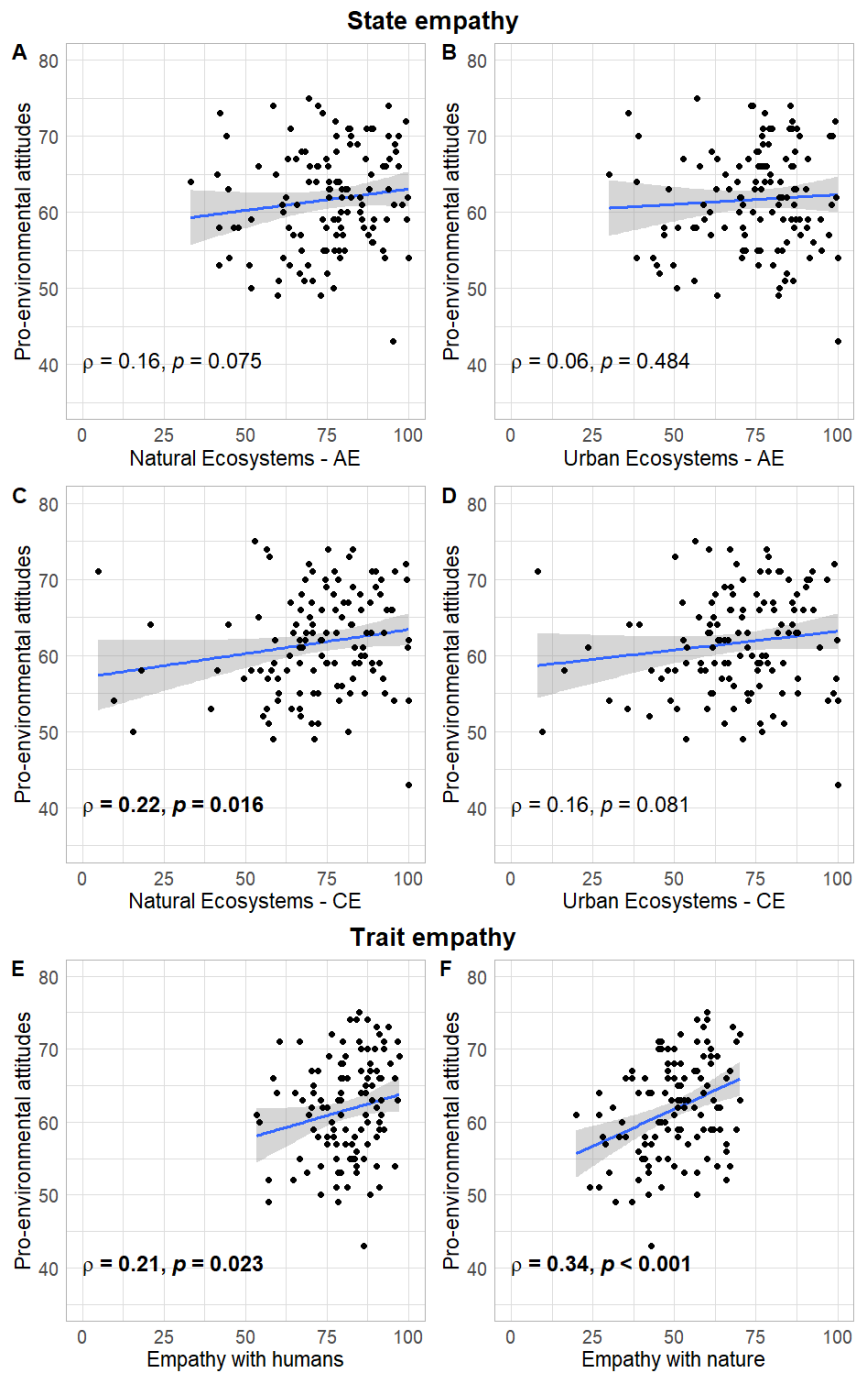
Scatter plots of the Spearman’s correlations between pro-environmental attitudes and state empathy with ecosystems, and trait empathy with nature and humans. (A) Correlations between state affective empathy with natural ecosystems (Natural Ecosystems—AE) and pro-environmental attitudes. (B) Correlations between state affective empathy with urban ecosystems (Urban Ecosystems—AE) and pro-environmental attitudes. (C) Correlations between state cognitive empathy with natural ecosystems (Natural ecosystems—CE) and pro-environmental attitudes. (D) Correlations between state cognitive empathy with urban ecosystems (Urban ecosystems—CE) and pro-environmental attitudes. (E) Correlations between trait empathy with humans and pro-environmental attitudes. (F) Correlations between trait empathy with nature and pro-environmental attitudes. Each dot represents a participant. The blue line indicates the tendency of the linear regression and the grey zone shows the 95% confidence interval. Spearman rho (*ρ*) and *p*-values (p) are provided for each correlation.

Pro-environmental attitudes were positively correlated with state affective empathy with animals (Spearman’s rho = 0.27, *p* = .003), state cognitive empathy with animals (Spearman’s rho = 0.26, *p* = .004) and state cognitive empathy with natural ecosystems (Spearman’s rho = 0.22, *p* = .016; Fig. 4C). Pro-environmental attitudes were not correlated with affective and cognitive state empathy with humans (*p* >.70 for all comparisons; Fig. 3; Table S10). All correlations remained significant after correction for multiple comparisons.

#### Correlations between trait empathy and pro-environmental attitudes

Pro-environmental attitudes exhibited positive, moderate and significant correlations with trait empathy with other-than-human beings (Spearman’s rho = 0.34, *p* < .001; Fig. 4E). Pro-environmental attitudes also correlate significantly and positively with empathy with humans (Spearman’s rho = 0.22, *p* = .016; Fig. 4F; Table S10). These correlations remained significant after correction for multiple comparison.

### Relations between empathy and human-nature connectedness

We found no significant correlations between state empathy measures and human-nature connectedness (Table S11). Trait empathy with other-than-human beings was moderately and positively correlated with human-nature connectedness (Spearman’s rho = 0.23, *p* = .012; Table S11). These correlations remained significant after correction for multiple comparison.

### Effects of demographical variables

We found a significant effect of sex on state affective and cognitive empathy. Men showed a lower state affective empathy than women only for the human and animal categories. We found no effect of sex for the natural ecosystems and urban ecosystems categories (Humans: *β* = 5.20, CI = [1.69–8.71], *p* = .004, Animals: *β* = −0.56, CI = [−1.10 to −0.03], *p* = .040; Natural Ecosystems: *β* = 2.43, CI = [3.63–8.49], *p* = .429; Urban Ecosystems: *β* = 6.10, CI = [−0.06–12.27], *p* = .052). Males show a lower state cognitive empathy than females for all categories (Humans: *β* = 6.52, CI = [0.09–12.95], *p* = .047, Animals: *β* = −0.83, CI = [−1.57 to −0.09], *p* = .028; Natural Ecosystems: *β* = 9.44, CI = [2.35–16.53], *p* = .010; Urban Ecosystems: *β* = 7.80, CI = [0.57–15.02], *p* = .035).

No effect of childhood environment was found on state affective empathy for all categories. However, childhood environment was found to have an effect on state cognitive empathy: individuals growing up in an urban environment show a significantly lower state cognitive empathy than individuals from a rural environment for all categories except for animals, for which no correlation was found (Humans: *β* = −10.32, CI = [−19.83 to −0.81], *p* = .001, Animals: *β* = 1.05, CI = [−0.05–2.14], *p* = .060; Natural Ecosystems: *β* =−12.77, CI = [−23.26 to −2.27], *p* < .018; Urban Ecosystems: *β* = −12.30, CI = [−22.99 to −1.62], *p* = .024). We found no effect of childhood environment on affective empathy (Tables S12–S15).

### Empathy ratings across image categories

For cognitive empathy, human images were rated slightly higher than animal images (*p* = .025), and natural ecosystem images were rated slightly higher than urban ecosystem images (*p* < .001). In contrast, both human and animal images elicited higher empathy than ecosystem images (*p* < .001). For affective empathy, no significant differences were observed between human and animal images (*p* = .235) or between natural and urban ecosystem images (*p* = .384), while human and animal images still evoked significantly greater empathy than ecosystem images (*p* < .001).

## Discussion

In the present study, we investigated individuals’ empathic responses to images depicting humans and other-than-human beings (animals and ecosystems) in distress. By measuring both state and trait empathy, we investigated whether the concept of empathy, which is usually directed toward humans or other-than-human animals, can be extended to urban and natural ecosystems. Additionally, we examined the relationship between state empathy with ecosystems and pro-environmental attitudes. We show that the concept of empathy can indeed be extended to ecosystems, at least within a population of French students represented in our sample. We further show that empathy with other-than-human beings is positively associated with pro-environmental attitudes.

Our findings reveal significant, moderate-to-strong correlations between empathy with humans and empathy with ecosystems in a sample of French students. This suggests that the behavioral, and likely neural basis for empathy extends beyond human relationships (Geiger et al., 2017), fostering a broader connection to the natural world. The strength of these associations suggests that individuals who are more attuned to the emotions of other humans may also be more sensitive to the experiences of non-human systems. These results align with theoretical perspectives proposing that empathic capacities extend beyond interpersonal contexts to broader ecological and environmental concerns, highlighting the potential for empathy-based interventions to promote pro-environmental behavior.

State cognitive empathy showed stronger cross-category associations—that is, higher correlations between empathic responses to different target types (humans, other-than-human animals, and ecosystems)—than state affective empathy. This pattern suggests that state cognitive empathy may rely on a category-independent mechanism, whereas state affective empathy might be more target-dependent and influenced by individual differences, resulting in weaker cross-category associations. Affective empathic responses to urban and natural ecosystems were strongly linked, suggesting a more unified emotional response to ecosystem-related suffering than to human or other-than-human animals suffering.

In line with previous studies (Sevillano, Corraliza & Lorenzo, 2017; Tam, 2013), trait empathy with humans was positively linked to trait empathy with nature, but to a lesser extent than the associations observed among state empathies. Because trait empathy reflects a stable tendency (*e.g.*, Davis, 1983), whereas state empathy captures momentary, situationally driven responses that vary depending on the stimulus presented (*e.g.*, Nezlek et al., 2007), the stronger correlations among state measures may be explained by shared situational variance. Specifically, all state empathy measures were elicited by similar distressing photographs, meaning that participants’ responses were influenced not only by their empathic disposition but also by common characteristics of the stimuli and the experimental context. This shared contextual influence likely increased the covariance among state measures. In contrast, trait empathy reflects broader dispositional tendencies that are less dependent on a specific situational context, resulting in weaker associations.

While both dimensions of state empathy with animals were linked to pro-environmental attitudes, only the cognitive dimension of state empathy with natural ecosystems was positively associated with such attitudes. This raises an interesting and somewhat counterintuitive point: emotional engagement with natural ecosystems, although it may evoke concern or sadness about the degradation of nature, does not necessarily translate into pro-environmental attitudes. Why is this?

One possible explanation is that the ability to understand environmental issues and perceive the complex relationships within ecosystems may have a more significant influence on developing pro-environmental attitudes than a purely emotional response to nature’s distress. For example, feeling negative emotions about animals’ pain might be relatively straightforward and sufficient to generate concern for their well-being, leading to pro-environmental attitudes without the need for a deep understanding of ecological systems. In contrast, emotional empathy with ecosystems in distress might not be enough to shift attitudes unless it is paired with a cognitive understanding of environmental issues and the necessity of taking action. These findings suggest that interventions aimed at fostering pro-environmental attitudes should combine affective engagement with information about environmental processes. For example, environmental programs could include activities that promote perspective-taking and emotional concern for nature alongside information on ecological interdependencies and human impacts. Such an integrated approach may be more effective than relying solely on emotional appeals to distress or concern for individual species.

Moreover, an affective empathic response might not be always appropriate to taking action. Intense situations of suffering can lead to *empathic overarousal* (Myers, Saunders & Bexell, 2009)—a form of emotional fatigue or disengagement (Hoffmann, 2008)—which is likely to prevent individuals to maintain long-term concern or act on their pro-environmental beliefs (Tam, 2013). Research in social psychological contexts has shown that individuals with higher affective empathic tendencies may experience greater anxiety when exposed to others’ distress, an effect referred to as *vicarious anxiety* (Shu et al., 2017). In addition, meta-analytic evidence (Nair et al., 2024) suggests that anxiety and affective components of empathy are modestly, but significantly, associated, indicating that heightened emotional resonance can coincide with increased anxiety. These findings support the idea that without effective emotion regulation, empathic overarousal could contribute to distress in response to environmental degradation, paralleling work on social anxiety where regulation strategies help mitigate negative emotional outcomes. Managing empathic overarousal may be crucial, as emerging work on the “Goldilocks” heuristic (Heeren & Clayton, 2026) suggests that climate-related anxiety is adaptive when maintained within an optimal range, whereas excessive arousal may lead to distress and disengagement rather than constructive action.

As expected, trait empathy with nature was moderately correlated with pro-environmental attitudes, as corroborated by other studies (Brown et al., 2019; Raymond et al., 2025; Tam, 2013). This association suggests that trait empathy with nature may predispose individuals to hold stronger pro-environmental attitudes. However, the moderate strength of this relationship indicates that trait empathy with nature is likely one contributing factor among others, rather than a sufficient determinant of pro-environmental attitudes.

Human-nature connectedness showed no link with state empathy but a positive link with trait empathy. We initially expected human-nature connectedness to function as a trait exerting a direct effect on state empathy. However, in our study, it seems to only relate to trait empathy with humans and other-than-human beings. One possible explanation is that, because the scale we used measures *trait* human-nature connectedness, it shows stronger correlations with trait empathy.

Another possible interpretation is that human-nature connectedness itself is not a sufficient trait to raise empathic responses, suggesting other personal processes to be the drivers of behavioral empathic processes, such as self-construal (Wang et al., 2023), which is not taken into account by our current questionnaire. Self-construal describes the degree to which an individual primarily feels independent from or interconnected to others (Giacomin & Jordan, 2017). Individuals with an independent self-construal tend to view themselves as independent entities with internal traits and values forming their individual uniqueness, whether individuals with an interdependent self-construal believe themselves to be related to others and view social groups or relationships as central. Only one study investigated the role of self-construal applied to empathy with nature (Wang et al., 2023). They found self-construal to act as a moderator between trait empathy with nature and pro-environmental behaviors. However, no measure of human-nature connectedness was included in this study, which does not allow us to compare the effects of these two elements on empathy with nature.

Moreover, our exploratory analysis aligns with well-established patterns in the empathy literature. As expected, women exhibited higher state cognitive empathy than men across all target categories, a trend consistently reported in psychological and environmental empathy research (Davis, 1983; Tam, 2013). This sex difference has often been interpreted through both socialization processes and gendered expectations regarding emotional expressiveness and care-oriented behavior.

Additionally, participants consistently rated images of humans and other-than-human animals higher in both cognitive and affective empathy than images of ecosystems. For cognitive empathy, small differences emerged—humans were rated slightly higher than animals, and natural ecosystems slightly higher than urban ecosystems—while no such differences appeared for affective empathy. These patterns align with theory and empirical evidence showing that empathic processes are more readily engaged by entities perceived as sentient agents than by non-sentient systems (*i.e.,* such as ecosystems; Batson et al., 1991; Preston & De Waal, 2002). Stronger responses toward humans and animals likely reflect cognitive and affective mechanisms prioritizing concern for beings perceived as capable of suffering, even though we did not assess participants’ knowledge or beliefs about the actual sentience of these other-than-human systems—something that could be explored in future studies.

Our exploratory results also indicate that individuals who grew up in city centers display lower state cognitive empathy toward nature compared with those raised in rural environments. Reduced direct contact with ecosystems in dense urban environments may limit opportunities to develop embodied, place-based experiences that support empathetic understanding-mechanisms also discussed by Brown et al. (2019) and in other research on the link between place attachment, or nature exposure, and pro-environmental dispositions (Chen, Tang & Liu, 2024; Swami et al., 2024; Whitburn, Linklater & Milfont, 2019). Together, these findings reinforce the notion that both social identities and childhood environments play a critical role in shaping cognitive empathy toward humans and the more-than-human world, suggesting an important role of education in the development of empathy with nature.

## Limitations and Future Studies

Several limitations must be acknowledged when interpreting our findings. Our study relies on a limited sample size in a non-representative part of the French population.

Moreover, the correlational design of the study limits the interpretation of the results. Future studies would benefit from replicating the experiment on variate socio-cultural populations.

Another limitation concerns the nature of the stimuli used to elicit state empathy. Incorporating a broader diversity of wild species, plants, and ecosystems would allow researchers to account for factors such as experience with the other-than-human beings presented (Decety, 2011), knowledge about such beings (Ienna et al., 2022), and similarity/closeness between human beings and the other-than-human beings presented (Geiger et al., 2017; Ingham, Neumann & Waters, 2015; Miralles, Raymond & Lecointre, 2019). Using more ecologically valid and emotionally informative stimuli may further enhance empathic responses: for example, Blythe et al. (2021) found that future scenarios, delivered *via* virtual reality or text, increased empathy with ocean, though effects diminished over time and depended on the emotional content. In contrast, Maruscakova et al. (2015) showed that human judgments of piglet vocalizations were driven primarily by acoustic features rather than empathy with humans or personality traits, highlighting that the features of the stimuli themselves can strongly shape empathic responses. Together, these findings suggest that future studies should carefully design stimuli that are diverse, ecologically valid, and emotionally informative to reliably elicit state empathy.

Similarly, more refined physiological measures, such as skin conductance (Westbury & Neumann, 2008), could help determine whether empathic arousal—the level of physiological activation associated with the strength of an emotional response—is linked to the declarative answers given by participants. In addition, facial expressions provide a complementary, objective measure of empathy that is less susceptible to social desirability bias or limitations of self-report. For instance, Ly & Weary (2021) demonstrated that human observers show reliable facial expressions of unpleasantness when viewing videos of farm animals undergoing painful procedures, and that the intensity of these expressions correlates with participants’ self-reported emotional responses. These findings suggest that combining physiological and behavioral measures, such as skin conductance and facial expressions, could provide a more complete and nuanced assessment of empathic responses, particularly in contexts where social desirability or introspective limitations might affect self-report data.

In addition, our measures of both trait and state empathy focused exclusively on suffering, based on the theoretical assumption that individuals are more aroused by distressing situations than by positive ones (Tam, 2013). However, empathic joy is an often-overlooked component of empathy with nature, and to our knowledge, is not captured by the only available measure specifically assessing dispositional empathy with nature (Tam, 2013). Regarding state empathic joy, most studies using undisturbed natural stimuli consider them as neutral in their experimental framework (Berenguer, 2007; Jing et al., 2022) and therefore do not discuss positive empathic responses. This raises the question of whether pro-environmental attitudes are also linked to empathic joy with nature, and including positive emotional components in future empathy scales would help clarify this issue.

The scale used to measure pro-environmental attitudes showed low internal consistency within our population. Therefore, the scale used, although validated in a previous study, may not be adapted to our specific population or to the context of our study (French students). This low reliability can be due to different factors, such as a maladaptation over time or contextual differences. Given these issues, we recommend some caution in using this scale in similar populations. Future research could benefit from revising the scale by adapting the items or relying on alternative scales with better psychometric properties and reliability, such as the long version developed by Milfont & Duckitt (2010).

Finally, we focused solely on pro-environmental attitudes, without measuring self-reported or actual sustainable behaviors. Pro-environmental behaviors can be measured through either declarative statements or objective indicators like eco-donations (Ibanez & Roussel, 2022; Lange, 2022; Lange & Dewitte, 2019). Future studies including a validated pro-environmental behaviors scale and effective behaviors are warranted.

Another potential limitation is the use of a one-item scale (Kleespies et al., 2021) to assess trait human-nature connectedness, which might have prevented us to capture the underlying mechanisms and the nuanced links between human-nature connectedness, state and trait empathy. A possible future study might include a measure of state human-nature connectedness—such as the state Connectedness with Nature Scale (CNS; Mayer et al., 2009) or the Emotional and Cognitive Scale of Human-Nature Relationships (ECS-HNR; Mundaca et al., 2021)—which would likely show stronger correlations with state empathy. However, the choice is limited as only a few developed scales consider human-nature connectedness as a state (Tiscareno-Osorno et al., 2023).

Our study highlights the role of cognitive empathy in pro-environmental attitudes. Building on these results, future research could examine whether interventions combining empathy training with knowledge-based or anthropomorphizing interventions are more effective than either alone, given evidence that environmental knowledge seems to remain a strong driver of behavior (Ienna et al., 2022). Virtual reality (VR) can be an effective tool for enhancing cognitive empathy in some contexts, as shown in studies reporting short-term increases in perspective-taking (Blythe et al., 2021). However, evidence from a recent meta-analysis (Martingano, Hererra & Konrath, 2021) suggests that for promoting lasting cognitive empathy toward the environment often requires an active engagement of, interventions that actively engage imaginative perspective-taking processes may be more consistently effective than fully passive and predetermined immersive experiences, for promoting lasting cognitive empathy toward the environment. This does not preclude the usefulness of VR, but highlights that its design and implementation should encourage active cognitive engagement rather than mere exposure.

Finally, as research increasingly highlights the role of cognitive empathy in shaping pro-environmental attitudes and ecological behaviors, future work should systematically explore how interventions targeting perspective-taking can be integrated into environmental education, policy campaigns, and conservation programs. One promising direction is to design longitudinal studies that assess whether enhancing cognitive empathy toward both humans and other-than-human communities leads to durable changes in conservation behavior, rather than the transient shifts often measured in laboratory settings. Moreover, future research should examine potential trade-offs and boundary conditions: for instance, recent work suggests that while trait cognitive empathy positively predicts conservation behavior, trait affective empathy may sometimes hinder action, especially in public contexts (Raymond et al., 2025).

## Conclusion

In conclusion, we show that humans’ empathy can be extended to other-than-human beings, including ecosystems. We also found that this broader empathy, and more specifically, state cognitive empathy, is linked to pro-environmental attitudes. By highlighting the key role of cognitive empathy, our findings suggest that activities aimed at taking the perspective of ecosystems or enhancing the understanding of ecosystems’ functioning may effectively promote pro-environmental attitudes and values. This insight challenges previous assumptions about the scope of empathy and provides a promising avenue for fostering greater environmental stewardship and addressing pressing sustainability issues.

##  Supplemental Information

10.7717/peerj.21383/supp-1 Supplemental Information 1 Supplemental Information - Exploratory Analyses

10.7717/peerj.21383/supp-2 Supplemental Information 2 Scatter plots of the Spearman’s correlations for affective (AE) and cognitive (CE) empathy related to perceived valence for all categories A) Correlations between affective empathy with humans (Humans –AE) and perceived valence. B) Correlations between affective empathy with animals (Animals –AE) and perceived valence. C) Correlations between affective empathy with natural ecosystems (Natural Ecosystems –AE) and perceived valence. D) Correlations between affective empathy with urban ecosystems (Urban Ecosystems –AE) and perceived valence. E) Correlations between cognitive empathy with humans (Humans –CE) and perceived valence. F) Correlations between cognitive empathy with animals (Animals –CE) and perceived valence. G) Correlations between cognitive empathy with natural ecosystems (Natural ecosystems –CE) and perceived valence. H) Correlations between cognitive empathy with urban ecosystems (Urban ecosystems –CE) and perceived valence. Each point represents a participant. The blue line represents the tendency of the linear regression and the grey zone represents the 95% confidence interval. Spearman rho (*ρ* ) and p-values (*p*) are provided for each correlation.

10.7717/peerj.21383/supp-3 Supplemental Information 3 Scatter plots of the Spearman’s correlations for state affective (AE) and cognitive (CE) empathy related to perceived arousal for all categories A) Correlations between affective empathy with humans (Humans –AE) and perceived arousal. B) Correlations between affective empathy with animals (Animals –AE) and perceived arousal. C) Correlations between affective empathy with natural ecosystems (Natural Ecosystems –AE) and perceived arousal. D) Correlations between affective empathy with urban ecosystems (Urban Ecosystems –AE) and perceived arousal. E) Correlations between cognitive empathy with humans (Humans –CE) and perceived arousal. F) Correlations between cognitive empathy with animals (Animals –CE) and perceived arousal. G) Correlations between cognitive empathy with natural ecosystems (Natural ecosystems –CE) and perceived arousal. H) Correlations between cognitive empathy with urban ecosystems (Urban ecosystems –CE) and perceived arousal. Each point represents a participant. The blue line represents the tendency of the linear regression and the grey zone represents the 95% confidence interval. Spearman rho (*ρ* ) and p-values (*p*) are provided for each correlation.

10.7717/peerj.21383/supp-4 Supplemental Information 4 Illustrated extension of the Inclusion of Nature in Self scale (Kleespies et al., 2021) 1 item. 7-points scale. Instructions were as follows: “In the next slide you’ll see a series of drawings representing your relationship with nature. You’ll see two circles represented. For each drawing, the circle containing a silhouette represents you (“Me”) and the circle containing a landscape represents nature (“Nature”). The circles overlap to a greater or lesser extent, depending on your relationship with nature: For example, if you choose A, it means you think there’s no connection between you and nature. On the other hand, if you choose G, it means you think you’re completely part of nature.” A score from 1 (A) to 7 (G) was computed for each participant.

10.7717/peerj.21383/supp-5 Supplemental Information 5 Scatter plots of the Spearman’s correlations for state affective (AE) and cognitive (CE) empathy related to trait empathy with nature A) Correlations between affective empathy with animals (Animals –AE) and trait empathy with nature. B) Correlations between affective empathy with natural ecosystems (Natural Ecosystems –AE) and trait empathy with nature. C) Correlations between affective empathy with urban ecosystems (Urban Ecosystems –AE) and trait empathy with nature. D) Correlations between cognitive empathy with animals (Animals –CE) and trait empathy with nature. E) Correlations between cognitive empathy with natural ecosystems (Natural ecosystems –CE) and trait empathy with nature. F) Correlations between cognitive empathy with urban ecosystems (Urban ecosystems –CE) and trait empathy with nature. Each point represents a participant. The blue line represents the tendency of the linear regression and the grey zone represents the 95% confidence interval. Spearman rho (*ρ* ) and p-values (*p*) are provided for each correlation.

10.7717/peerj.21383/supp-6 Supplemental Information 6 Scatter plots of the Spearman’s correlations of trait empathy with humans and nature, their affective and (AE) and cognitive (CE) subscores related to pro-environmental attitudes A) Correlations between global score of trait empathy with humans and pro-environmental attitudes. B) Correlations between affective subscore of empathy with humans (Humans –AE subscore) and pro-environmental attitudes. C) Correlations between cognitive subscore of empathy with humans (Humans –CE subscore) and pro-environmental attitudes. D) Correlations between global score of trait empathy with nature and pro-environmental attitudes. E) Correlations between affective subscore of empathy with nature (Nature –AE subscore) and pro-environmental attitudes. F) Correlations between cognitive subscore of empathy with nature (Nature –CE subscore) and pro-environmental attitudes. Each point represents a participant. The blue line represents the tendency of the linear regression and the grey zone represents the 95% confidence interval. Spearman rho (*ρ* ) and p-values (*p*) are provided for each correlation.

10.7717/peerj.21383/supp-7 Supplemental Information 7 Demographical repartition of the participants

10.7717/peerj.21383/supp-8 Supplemental Information 8 Valence and arousal response per category The databases (Dan-Glauser & Scherer, 2011; Kurdi, Lozano & Banaji, 2017; Ottavi, Roussel & Syssau, 2021) from which each image was extracted are indicated below each picture. Valence and arousal values were rescaled on a 0 to 100-points scale.

10.7717/peerj.21383/supp-9 Supplemental Information 9 Affective and Cognitive Measure of Empathy (ACME) scale (Savard et al., 2022) 36 items on a 5-points scale. (R) indicates the item is reverse scored. CE stands for Cognitive Empathy and AE for Affective Empathy. Instruction reads: “Using the indications below, indicate to what extent you Agree or Disagree with each of the following statements. Give only one answer for each proposition.”

10.7717/peerj.21383/supp-10 Supplemental Information 10 Dispositional empathy with nature scale (DENS) (Tam, 2013) 10 items on a 7-points scale. The instruction of the DENS reads: “Nowadays, we often hear news reporting how nature is being destroyed by humans. For instance, rivers are being polluted by chemicals or toxic waste from factories, oceans being polluted by deep-water oil spill, forests being cleared and degraded into wasteland. Many animals and plants living in nature are suffering. We want to know how you think and feel when you hear this type of news. According to this scale (*1=strongly disagree; 2=disagree; 3=mildly disagree; 4=neither disagree or agree; 5=mildly agree; 6=agree; 7=strongly agree*), please write a number before each item to indicate your agreement or disagreement with it”. CE: Items reflecting cognitive empathy; AE: items reflecting affective empathy.

10.7717/peerj.21383/supp-11 Supplemental Information 11 Environmental Attitudes Inventory (Milfont & Duckitt, 2010; Moussaoui et al., 2016) 12 items on a 7-points scale. (R) indicates the item is reverse scored. Instruction reads: “Please indicate your choice by ticking the appropriate number. There are no right or wrong answers. Do not dwell too long on the statements. 7-point response scale: 1: totally disagree - 7: totally agree”

10.7717/peerj.21383/supp-12 Supplemental Information 12 Social desirability scale (Crowne & Marlowe, 1960); Sârbescu et al., 2012) 13 items on a True/False scale. Instruction reads as follows: “Listed below are a number of statements concerning personal attitudes and traits. Read each item and decide whether the statement is true or false as it pertains to you personally.”

10.7717/peerj.21383/supp-13 Supplemental Information 13 Distribution of key variables AE stands for Affective Empathy and CE for Cognitive Empathy.

10.7717/peerj.21383/supp-14 Supplemental Information 14 Results of the 2 (empathy type: cognitive, affective) × 4 (image category) repeated-measures ANOVA AE indicates participants’ values of behavioral affective empathy and CE, values of behavioral cognitive empathy. Significant results are shown in bold.

10.7717/peerj.21383/supp-15 Supplemental Information 15 Correlations between quantitative measures of state and trait empathy Spearman’s rho correlation coefficients. Significant results appear in black and non-significant results in light grey. Numerical p-values are written below each Spearman’s correlation coefficient. AE stands for Affective Empathy and CE for Cognitive Empathy. Trait empathy with humans corresponds to the ACME scale (Table S3) and trait empathy with nature corresponds to the DEN scale (Table S4). Values that were not significant after correction for multiple comparison appear in orange.

10.7717/peerj.21383/supp-16 Supplemental Information 16 Correlations of state and trait empathy measures with pro-environmental attitudes Spearman’s rho correlation coefficients. Significant results appear in black and non-significant results in light grey. Numerical p-values are written below each Spearman’s correlation coefficient. AE stands for Affective Empathy and CE for Cognitive Empathy. Trait empathy with humans corresponds to the ACME scale (Table S3) and trait empathy with nature corresponds to the DEN scale (Table S4). Pro-environmental attitudes correspond to the EAI scale (Table S5). No change in significance was observed after correction for multiple comparison.

10.7717/peerj.21383/supp-17 Supplemental Information 17 Correlations of state and trait empathy measures with human-nature connectedness Spearman’s rho correlation coefficients. Significant results appear in black and non-significant results in light grey. Numerical p-values are written below each Spearman’s correlation coefficient. AE stands for Affective Empathy and CE for Cognitive Empathy. Trait empathy with humans corresponds to the ACME scale (Table S3) and trait empathy with nature corresponds to the DEN scale (Table S4). Human-Nature Connectedness correspond to the IINS scale (Fig. S2). No change in significance was observed after correction for multiple comparison.

10.7717/peerj.21383/supp-18 Supplemental Information 18 Effects of demographical variables on behavioral empathy for the human’s pictures category Models were obtained using the lm() function of the R language, aimed to fit linear models to datasets. AE stands for Affective Empathy, CE for Cognitive Empathy and HNC for Human-Nature Connectedness.

10.7717/peerj.21383/supp-19 Supplemental Information 19 Effects of demographical variables on behavioral empathy for the animal’s pictures category Models were obtained using the lm() function of the R language, aimed to fit linear models to datasets. Note: due to the normalization applied to these variables, negative estimates represent a positive effect of the variables. AE stands for Affective Empathy, CE for Cognitive Empathy and HNC for Human-Nature Connectedness.

10.7717/peerj.21383/supp-20 Supplemental Information 20 Effects of demographical variables on behavioral empathy for the natural ecosystem’s picture category Models were obtained using the lm() function of the R language, aimed to fit linear models to datasets. AE stands for Affective Empathy, CE for Cognitive Empathy and HNC for Human-Nature Connectedness.

10.7717/peerj.21383/supp-21 Supplemental Information 21 Effects of demographical variables on behavioral empathy for the urban ecosystem’s pictures category Models were obtained using the lm() function of the R language, aimed to fit linear models to datasets. AE stands for Affective Empathy, CE for Cognitive Empathy and HNC for Human-Nature Connectedness.

10.7717/peerj.21383/supp-22 Supplemental Information 22 Supplemental code and data

## Additional Information and Declarations

### Data Availability

The following information was supplied regarding data availability: The raw data and code are available in the Supplemental Files.
