# Supplementary material for: Nature can suffer, too: behavioral evidence of empathy with ecosystems and its link to pro-environmental attitudes
Source: PeerJ. 2026 Jun 26;14:e21383. doi: 10.7717/peerj.21383 (PMC13312967; doi:10.7717/peerj.21383)
Supplement: Supplemental Information 1 [file peerj-14-21383-s001.pdf]

Supplemental Information Text

Exploratory analyses

Extended methods

Since six items reflected the affective dimension (empathic concern) and four items the cognitive dimension (perspective taking), we also calculated sub-scores for trait affective empathy with nature (AEN, $\alpha = 0.83$) and trait cognitive empathy with nature (CEN, $\alpha = 0.82$) for exploratory analyses.

Extended results

Correlations between trait empathy measures

Trait affective empathy toward humans was positively significantly correlated with trait affective empathy toward nature subscore (Spearman's rho = 0.26, $p = .004$). Similarly, trait cognitive empathy toward humans was positively significantly correlated with trait cognitive empathy toward nature subscore (Spearman's rho = 0.23, $p = .012$).

Correlations between trait empathy and pro-environmental attitudes

Pro-environmental attitudes also exhibited positive and moderate correlations with trait cognitive empathy toward humans (Spearman's rho = 0.22, $p = .015$; Fig. 6C), trait affective empathy toward nature (Spearman's rho = 0.28, $p = .002$; Fig. 6E) and trait cognitive empathy toward nature (Spearman's rho = 0.34, $p < .001$; Fig. 6F), but not with trait affective and trait cognitive empathy toward humans (Fig. 6B).

Correlation between trait empathy and human-nature connectedness

We found a positive and moderate correlation between trait *empathy with humans* and human-nature connectedness (Spearman's rho = 0.36, $p < .001$). Specifically, trait affective empathy with humans and trait cognitive empathy with humans were positively correlated with human-nature connectedness (Spearman's rho = 0.33, $p < .001$ and Spearman's rho = 0.29, $p < .001$, respectively).

More specifically, trait affective empathy with nature as well as trait cognitive empathy with nature showed significant positive relationship with human-nature connectedness (Spearman's rho = 0.20, $p = .028$ and Spearman's rho = 0.19, $p = .038$).
