# Supplementary figures and images for: Nature can suffer, too: behavioral evidence of empathy with ecosystems and its link to pro-environmental attitudes
Source: PeerJ. 2026 Jun 26;14:e21383. doi: 10.7717/peerj.21383 (PMC13312967; doi:10.7717/peerj.21383)

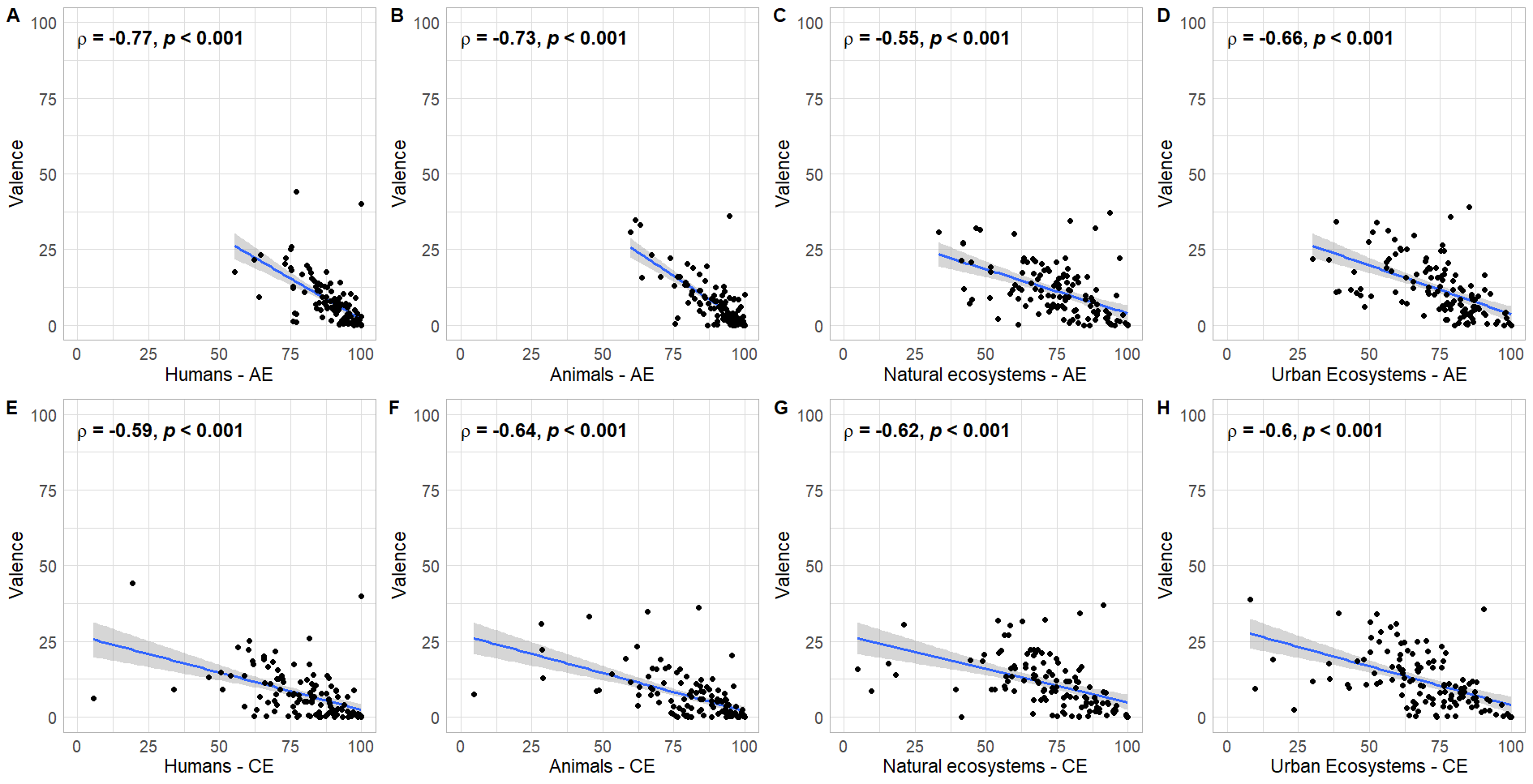

Supplement: Supplemental Information 2 — A) Correlations between affective empathy with humans (Humans –AE) and perceived valence. B) Correlations between affective empathy with animals (Animals –AE) and perceived valence. C) Correlations between affective empathy with natural ecosystems (Natural Ecosystems –AE) and perceived valence. D) Correlations between affective empathy with urban ecosystems (Urban Ecosystems –AE) and perceived valence. E) Correlations between cognitive empathy with humans (Humans –CE) and perceived valence. F) Correlations between cognitive empathy with animals (Animals –CE) and perceived valence. G) Correlations between cognitive empathy with natural ecosystems (Natural ecosystems –CE) and perceived valence. H) Correlations between cognitive empathy with urban ecosystems (Urban ecosystems –CE) and perceived valence. Each point represents a participant. The blue line represents the tendency of the linear regression and the grey zone represents the 95% confidence interval. Spearman rho (ρ ) and p-values (p) are provided for each correlation. [file peerj-14-21383-s002.png]

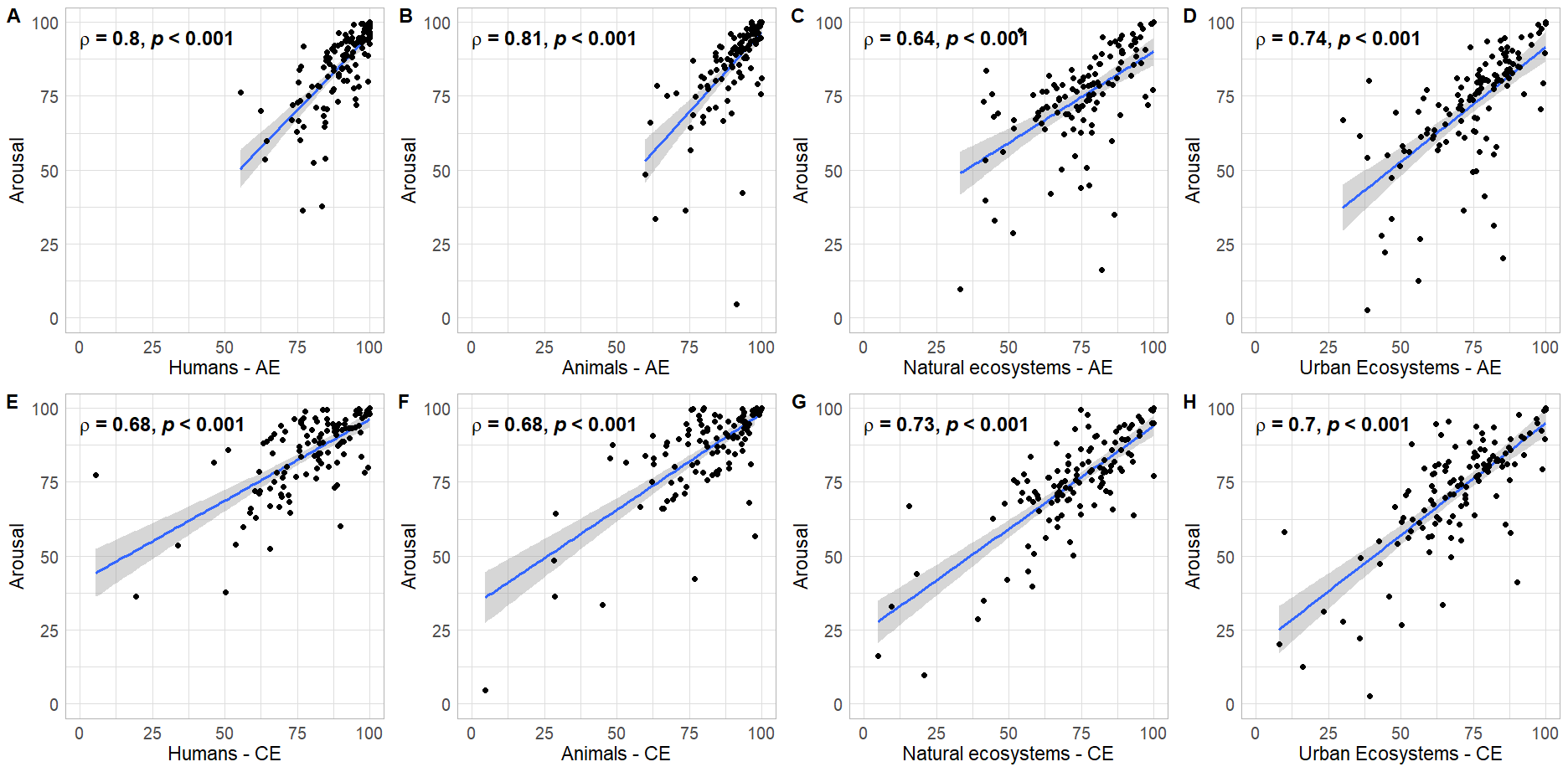

Supplement: Supplemental Information 3 — A) Correlations between affective empathy with humans (Humans –AE) and perceived arousal. B) Correlations between affective empathy with animals (Animals –AE) and perceived arousal. C) Correlations between affective empathy with natural ecosystems (Natural Ecosystems –AE) and perceived arousal. D) Correlations between affective empathy with urban ecosystems (Urban Ecosystems –AE) and perceived arousal. E) Correlations between cognitive empathy with humans (Humans –CE) and perceived arousal. F) Correlations between cognitive empathy with animals (Animals –CE) and perceived arousal. G) Correlations between cognitive empathy with natural ecosystems (Natural ecosystems –CE) and perceived arousal. H) Correlations between cognitive empathy with urban ecosystems (Urban ecosystems –CE) and perceived arousal. Each point represents a participant. The blue line represents the tendency of the linear regression and the grey zone represents the 95% confidence interval. Spearman rho (ρ ) and p-values (p) are provided for each correlation. [file peerj-14-21383-s003.png]

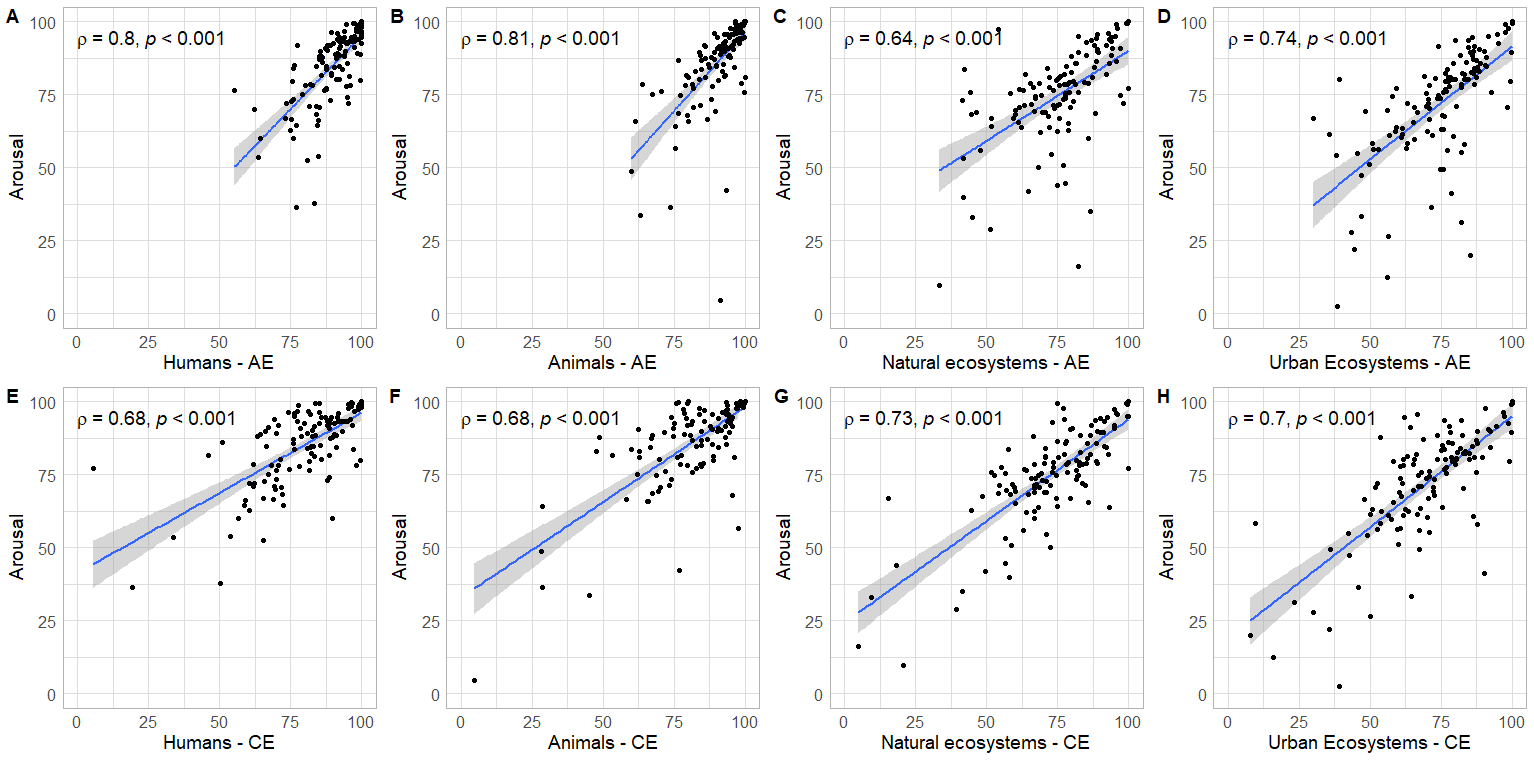

Supplement: Supplemental Information 4 — 1 item. 7-points scale. Instructions were as follows: “In the next slide you’ll see a series of drawings representing your relationship with nature. You’ll see two circles represented. For each drawing, the circle containing a silhouette represents you (“Me”) and the circle containing a landscape represents nature (“Nature”). The circles overlap to a greater or lesser extent, depending on your relationship with nature: For example, if you choose A, it means you think there’s no connection between you and nature. On the other hand, if you choose G, it means you think you’re completely part of nature.” A score from 1 (A) to 7 (G) was computed for each participant. [file peerj-14-21383-s004.png]

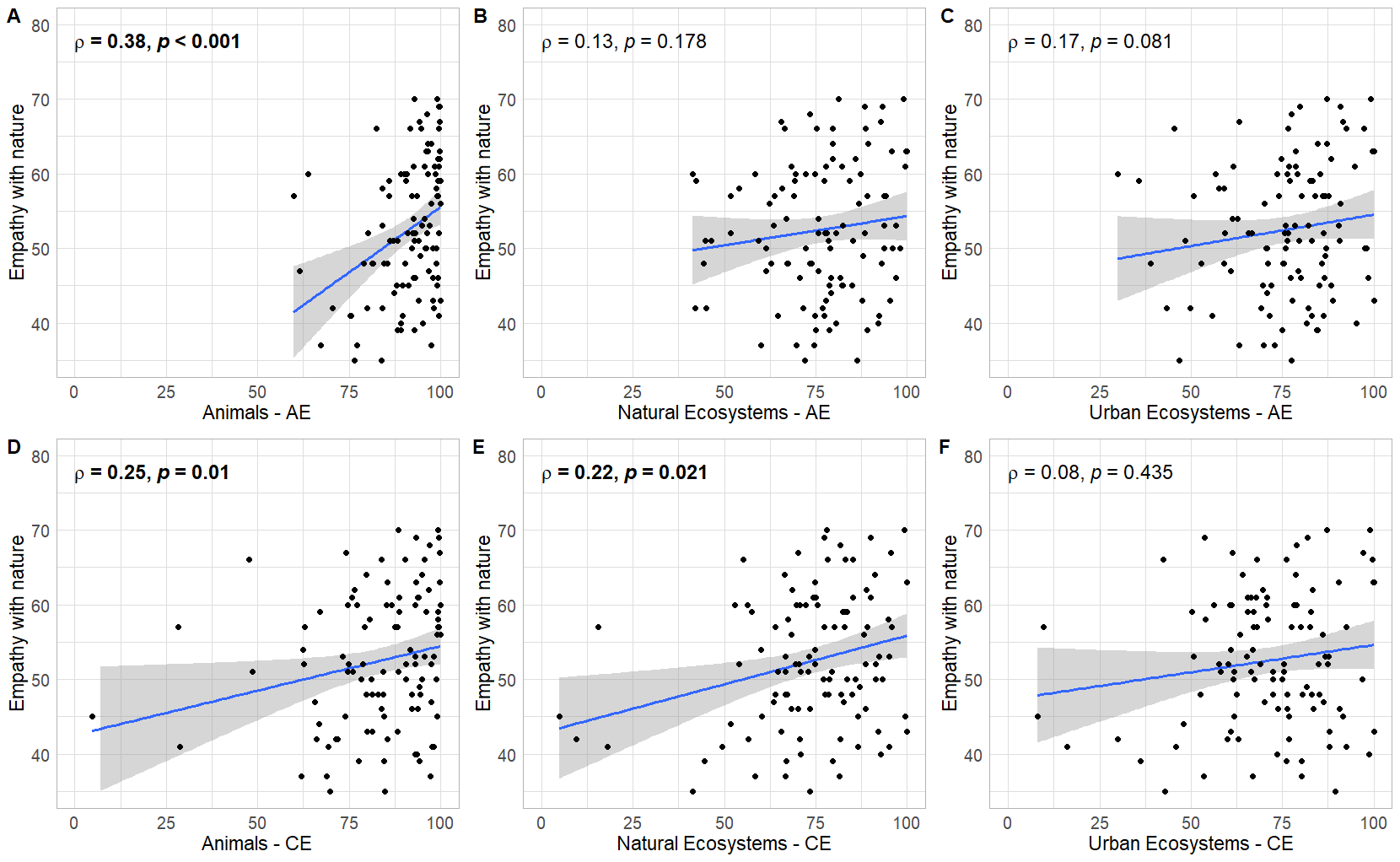

Supplement: Supplemental Information 5 — A) Correlations between affective empathy with animals (Animals –AE) and trait empathy with nature. B) Correlations between affective empathy with natural ecosystems (Natural Ecosystems –AE) and trait empathy with nature. C) Correlations between affective empathy with urban ecosystems (Urban Ecosystems –AE) and trait empathy with nature. D) Correlations between cognitive empathy with animals (Animals –CE) and trait empathy with nature. E) Correlations between cognitive empathy with natural ecosystems (Natural ecosystems –CE) and trait empathy with nature. F) Correlations between cognitive empathy with urban ecosystems (Urban ecosystems –CE) and trait empathy with nature. Each point represents a participant. The blue line represents the tendency of the linear regression and the grey zone represents the 95% confidence interval. Spearman rho (ρ ) and p-values (p) are provided for each correlation. [file peerj-14-21383-s005.png]

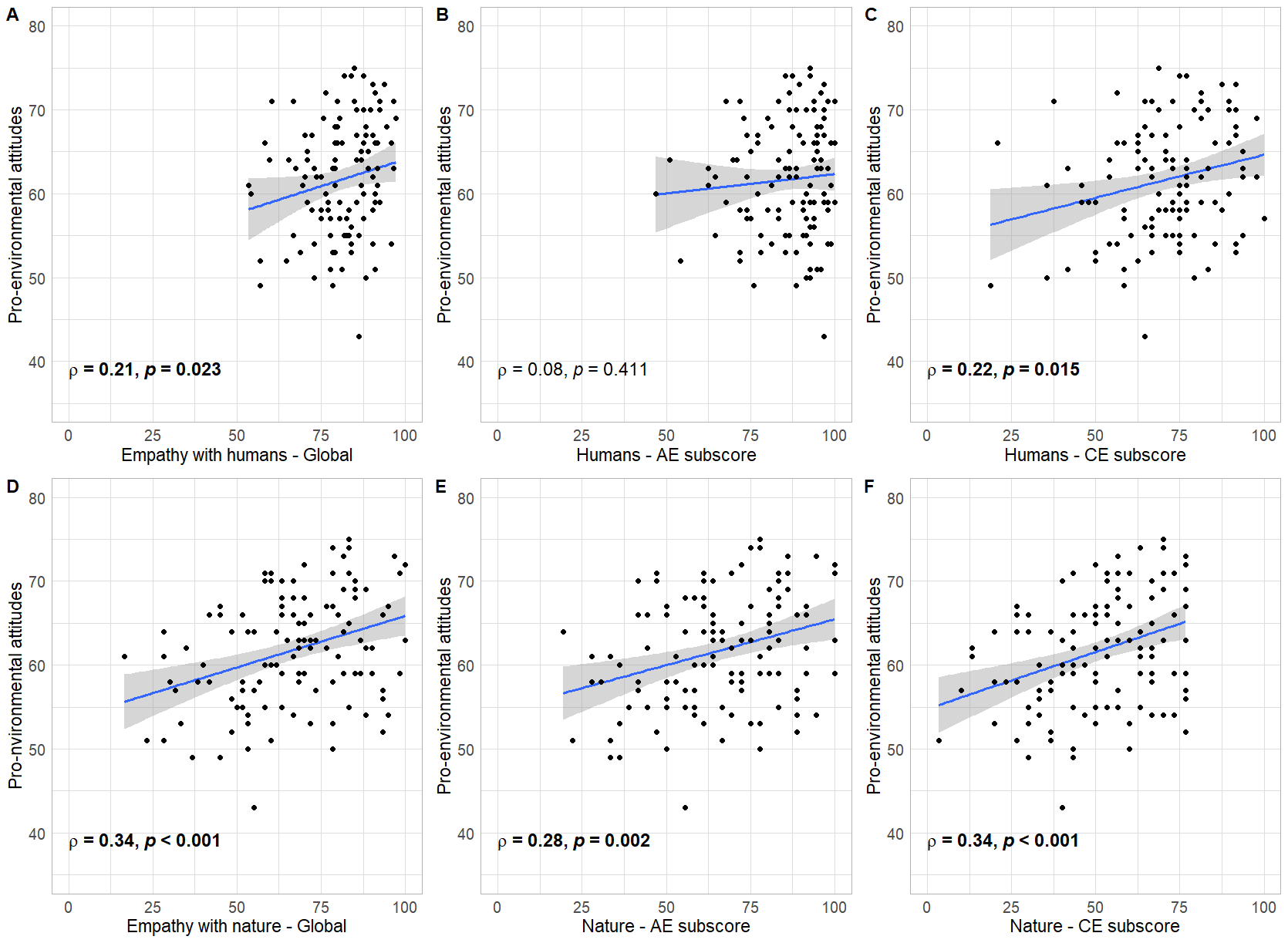

Supplement: Supplemental Information 6 — A) Correlations between global score of trait empathy with humans and pro-environmental attitudes. B) Correlations between affective subscore of empathy with humans (Humans –AE subscore) and pro-environmental attitudes. C) Correlations between cognitive subscore of empathy with humans (Humans –CE subscore) and pro-environmental attitudes. D) Correlations between global score of trait empathy with nature and pro-environmental attitudes. E) Correlations between affective subscore of empathy with nature (Nature –AE subscore) and pro-environmental attitudes. F) Correlations between cognitive subscore of empathy with nature (Nature –CE subscore) and pro-environmental attitudes. Each point represents a participant. The blue line represents the tendency of the linear regression and the grey zone represents the 95% confidence interval. Spearman rho (ρ ) and p-values (p) are provided for each correlation. [file peerj-14-21383-s006.png]
