# Supplementary material for: Nature can suffer, too: behavioral evidence of empathy with ecosystems and its link to pro-environmental attitudes
Source: PeerJ. 2026 Jun 26;14:e21383. doi: 10.7717/peerj.21383 (PMC13312967; doi:10.7717/peerj.21383)
Supplement: Supplemental Information 7 [file peerj-14-21383-s007.pdf]

Table S1. Demographical repartition of the participants.

	Overall (N=122)
**Age**	
Mean (SD)	21.6 (4.82)
Median [Min, Max]	20.0 [17.0, 50.0]
**Sex**	
Male	40 (32.8%)
Female	81 (66.4%)
Other	1 (0.8%)
**Education**	
School certificate	0 (0%)
High school diploma	9 (7.4%)
Bachelor's degree	96 (78.7%)
Master's degree	15 (12.3%)
PhD	1 (0.8%)
Other	1 (0.8%)
**Origin**	
Rural	30 (24.6%)
Semi-rural	37 (30.3%)
Urban	32 (26.2%)
City-center	23 (18.9%)
