# Supplementary material for: Nature can suffer, too: behavioral evidence of empathy with ecosystems and its link to pro-environmental attitudes
Source: PeerJ. 2026 Jun 26;14:e21383. doi: 10.7717/peerj.21383 (PMC13312967; doi:10.7717/peerj.21383)
Supplement: Supplemental Information 8 — The databases (Dan-Glauser & Scherer, 2011; Kurdi, Lozano & Banaji, 2017; Ottavi, Roussel & Syssau, 2021) from which each image was extracted are indicated below each picture. Valence and arousal values were rescaled on a 0 to 100-points scale. [file peerj-14-21383-s008.pdf]

Table S2. Valence and arousal response per category. The databases (Dan-Glauser & Scherer, 2011; Kurdi et al., 2017; Ottavi et al., 2021) from which each image was extracted are indicated below each picture. Valence and arousal values were rescaled on a 0 to 100-points scale.

**Cat.**	**Valence** mean (SD)	**Arousal** mean (SD)
Humans	18.6 (22.6)	61.2 (27.6)
Animals	13.9 (20.7)	69.2 (26.6)
Natural Ecosystems	11.5 (13.6)	70.1 (26.1)
Urban Ecosystems	9.0 (11.8)	68.8 (21.5)
