# Supplementary material for: Nature can suffer, too: behavioral evidence of empathy with ecosystems and its link to pro-environmental attitudes
Source: PeerJ. 2026 Jun 26;14:e21383. doi: 10.7717/peerj.21383 (PMC13312967; doi:10.7717/peerj.21383)
Supplement: Supplemental Information 9 — 36 items on a 5-points scale. (R) indicates the item is reverse scored. CE stands for Cognitive Empathy and AE for Affective Empathy. Instruction reads: “Using the indications below, indicate to what extent you Agree or Disagree with each of the following statements. Give only one answer for each proposition.” [file peerj-14-21383-s009.pdf]

Table S3. Affective and Cognitive Measure of Empathy (ACME) scale (Savard et al., 2022) – 36 items. 5-points scale. (R) indicates the item is reverse scored. CE stands for Cognitive Empathy and AE for Affective Empathy. Instruction reads: **"**Using the indications below, indicate to what extent you Agree or Disagree with each of the following statements. Give only one answer for each proposition.**"**

		Empathy Scale	Strongly disagree						Strongly agree
1	I have a hard time reading people's emotions.	CE	(R)	1	2	3	4	5	
2	I think it's fun to push people around once and a while.	AE	(R)	1	2	3	4	5	
3	I can tell when someone is afraid.	CE		1	2	3	4	5	
4	It's obvious when people are pretending to be happy.	CE		1	2	3	4	5	
5	I love watching people get angry.	AE	(R)	1	2	3	4	5	
6	I enjoy seeing strangers get scared.	AE	(R)	1	2	3	4	5	
7	It makes me feel good to help someone in need.	AE		1	2	3	4	5	
8	I get excited to give someone a gift that I think they will enjoy.	AE		1	2	3	4	5	
9	I usually understand why people feel the way they do.	CE		1	2	3	4	5	
10	When my friends are having a good time I often get angry.	AE	(R)	1	2	3	4	5	
11	People who are cheery disgust me.	AE	(R)	1	2	3	4	5	
12	I don't worry much about hurting people's feelings.	AE	(R)	1	2	3	4	5	
13	I don't really care if other people feel happy.	AE	(R)	1	2	3	4	5	
14	I have a hard time figuring out what someone else is feeling.	CE	(R)	1	2	3	4	5	
15	I can tell when people are about to lose their temper.	CE		1	2	3	4	5	
16	I can usually predict how someone will feel.	CE		1	2	3	4	5	
17	I don't really care if people are feeling depressed.	AE	(R)	1	2	3	4	5	
18	I like making other people uncomfortable.	AE	(R)	1	2	3	4	5	
19	I get a kick out of making other people feel stupid.	AE	(R)	1	2	3	4	5	
20	When my friends get angry I often feel like laughing.	AE	(R)	1	2	3	4	5	
21	Sometimes I enjoy seeing people cry.	AE	(R)	1	2	3	4	5	
22	Other people's feelings don't bother me at all.	AE	(R)	1	2	3	4	5	
23	I feel awful when I hurt someone's feelings.	AE		1	2	3	4	5	
24	Other people's misfortunes don't bother me much.	AE	(R)	1	2	3	4	5	
25	I can usually tell how people are feeling.	CE		1	2	3	4	5	
26	Sometimes it's funny to see people get humiliated.	AE	(R)	1	2	3	4	5	
27	If I could get away with it, there are some people I would enjoy hurting.	AE	(R)	1	2	3	4	5	
28	If I see that I am doing something that hurts someone, I will quickly stop.	AE		1	2	3	4	5	
29	I often try to help people feel better when they are upset.	AE		1	2	3	4	5	
30	I enjoy making others happy.	AE		1	2	3	4	5	
31	I am not good at understanding other people's emotions.	CE	(R)	1	2	3	4	5	
32	People have told me that I'm insensitive.	AE	(R)	1	2	3	4	5	
33	I can usually guess what's making someone angry.	CE		1	2	3	4	5	
34	People don't have to tell me when they're sad, I can see it in their faces.	CE		1	2	3	4	5	
35	I find it hard to tell when someone is sad.	CE	(R)	1	2	3	4	5	
36	I admit that I enjoy irritating other people.	AE	(R)	1	2	3	4	5	
