# Supplementary material for: Nature can suffer, too: behavioral evidence of empathy with ecosystems and its link to pro-environmental attitudes
Source: PeerJ. 2026 Jun 26;14:e21383. doi: 10.7717/peerj.21383 (PMC13312967; doi:10.7717/peerj.21383)
Supplement: Supplemental Information 10 — 10 items on a 7-points scale. The instruction of the DENS reads: “Nowadays, we often hear news reporting how nature is being destroyed by humans. For instance, rivers are being polluted by chemicals or toxic waste from factories, oceans being polluted by deep-water oil spill, forests being cleared and degraded into wasteland. Many animals and plants living in nature are suffering. We want to know how you think and feel when you hear this type of news. According to this scale (1=strongly disagree; 2=disagree; 3=mildly disagree; 4=neither disagree or agree; 5=mildly agree; 6=agree; 7=strongly agree), please write a number before each item to indicate your agreement or disagreement with it”. CE: Items reflecting cognitive empathy; AE: items reflecting affective empathy. [file peerj-14-21383-s010.pdf]

Table S4. Dispositional empathy with nature scale (DENS) (Tam, 2013) – 10 items. 7-points scale. The instruction of the DENS reads: "Nowadays, we often hear news reporting how nature is being destroyed by humans. For instance, rivers are being polluted by chemicals or toxic waste from factories, oceans being polluted by deep-water oil spill, forests being cleared and degraded into wasteland. Many animals and plants living in nature are suffering. We want to know how you think and feel when you hear this type of news. According to this scale (*1=strongly disagree; 2=disagree; 3=mildly disagree; 4=neither disagree or agree; 5=mildly agree; 6=agree; 7=strongly agree*), please write a number before each item to indicate your agreement or disagreement with it". CE: Items reflecting cognitive empathy; AE: items reflecting affective empathy.

		Empathy Scale	Strongly disagree							Strongly agree
1	I imagine how I would feel if I were the suffering animals and plants.	CE	1	2	3	4	5	6	7	
2	I get involved with the feelings of the suffering animals and plants.	AE	1	2	3	4	5	6	7	
3	I feel as though I were one of the suffering animals and plants.	AE	1	2	3	4	5	6	7	
4	I can very easily put myself in the place of the suffering animals and plants.	CE	1	2	3	4	5	6	7	
5	I try to understand how the suffering animals and plants feel by imagining how things look from their perspective.	CE	1	2	3	4	5	6	7	
6	I visualize in my mind clearly and vividly how the suffering animals and plants feel in their situation.	CE	1	2	3	4	5	6	7	
7	I have tender, concerned feelings for the suffering animals and plants.	AE	1	2	3	4	5	6	7	
8	I feel what the suffering animals and plants are feeling.	AE	1	2	3	4	5	6	7	
9	I feel the pain the suffering animals and plants are experiencing.	AE	1	2	3	4	5	6	7	
10	I feel sympathetic toward the suffering animals and plants.	AE	1	2	3	4	5	6	7	
