# Supplementary material for: Nature can suffer, too: behavioral evidence of empathy with ecosystems and its link to pro-environmental attitudes
Source: PeerJ. 2026 Jun 26;14:e21383. doi: 10.7717/peerj.21383 (PMC13312967; doi:10.7717/peerj.21383)
Supplement: Supplemental Information 11 — 12 items on a 7-points scale. (R) indicates the item is reverse scored. Instruction reads: “Please indicate your choice by ticking the appropriate number. There are no right or wrong answers. Do not dwell too long on the statements. 7-point response scale: 1: totally disagree - 7: totally agree” [file peerj-14-21383-s011.pdf]

Table S5. Environmental Attitudes Inventory (Milfont & Duckitt, 2010; Moussaoui et al., 2016) – 12 items – 7-points scale. (R) indicates the item is reverse scored. Instruction reads: "Please indicate your choice by ticking the appropriate number. There are no right or wrong answers. Do not dwell too long on the statements. 7-point response scale: 1: totally disagree - 7: totally agree"

		Strongly disagree								Strongly agree
1	I find that spending time in nature is boring.	(R)	1	2	3	4	5	6	7	
2	I'm opposed to the idea of governments controlling and regulating the use of raw materials in order to make them last longer.	(R)	1	2	3	4	5	6	7	
3	I would like to join and actively participate in an environmental group.		1	2	3	4	5	6	7	
4	We need to keep our lakes and rivers clean to protect the environment, and not to allow people to enjoy water sports		1	2	3	4	5	6	7	
5	Modern science will solve our environmental problems.	(R)	1	2	3	4	5	6	7	
6	Human beings exploit the environment too much.		1	2	3	4	5	6	7	
7	I prefer a wild, natural garden to a neat, well-ordered one.		1	2	3	4	5	6	7	
8	I'm not the kind of person who makes an effort to save natural resources	(R)	1	2	3	4	5	6	7	
9	Human beings were created or evolved to dominate the rest of nature.	(R)	1	2	3	4	5	6	7	
10	Protecting the environment is more important than protecting people's jobs		1	2	3	4	5	6	7	
11	It makes me sad seeing forests destroyed for agriculture.		1	2	3	4	5	6	7	
12	A married couple should be able to have as many children as they want, as long as they can provide for them properly.	(R)	1	2	3	4	5	6	7	
