# Supplementary material for: Nature can suffer, too: behavioral evidence of empathy with ecosystems and its link to pro-environmental attitudes
Source: PeerJ. 2026 Jun 26;14:e21383. doi: 10.7717/peerj.21383 (PMC13312967; doi:10.7717/peerj.21383)
Supplement: Supplemental Information 12 — 13 items on a True/False scale. Instruction reads as follows: “Listed below are a number of statements concerning personal attitudes and traits. Read each item and decide whether the statement is true or false as it pertains to you personally.” [file peerj-14-21383-s012.pdf]

Table S6. Social desirability scale (Crowne & Marlowe, 1960; Sârbescu et al., 2012) – 13 items – True/False scale. Instruction reads as follows: “Listed below are a number of statements concerning personal attitudes and traits. Read each item and decide whether the statement is true or false as it pertains to you personally.”

		Scores 0 if	Scores 1 if
1	I have never intensely disliked anyone.	False	True
2	I sometimes feel resentful when I don't get my way.	True	False
3	I like to gossip at times.	True	False
4	There have been times when I felt like rebelling against people in authority even though I knew they were right.	True	False
5	I can remember "playing sick" to get out of something.	True	False
6	There have been occasions when I took advantage of someone.	True	False
7	I sometimes try to get even rather than forgive and forget.	True	False
8	I am always courteous, even to people who are disagreeable.	False	True
9	There have been occasions when I felt like smashing things.	True	False
10	I have never been irked when people expressed ideas very different from my own.	False	True
11	There have been times when I was quite jealous of the good fortune of others.	True	False
12	I sometimes think when people have a misfortune, they only got what they deserved.	True	False
13	I have never deliberately said something that hurt someone's feelings.	False	True
