# Supplementary material for: Nature can suffer, too: behavioral evidence of empathy with ecosystems and its link to pro-environmental attitudes
Source: PeerJ. 2026 Jun 26;14:e21383. doi: 10.7717/peerj.21383 (PMC13312967; doi:10.7717/peerj.21383)
Supplement: Supplemental Information 13 — AE stands for Affective Empathy and CE for Cognitive Empathy. [file peerj-14-21383-s013.pdf]

Table S7. Distribution of key variables. AE stands for Affective Empathy and CE for Cognitive Empathy.

	D	*p*	Skewness	Kurtosis
Human AE	0.13	***0.02***	-1.15	4.27
Animal AE	0.16	***0.01***	-1.39	4.67
Natural Eco. AE	0.08	*0.44*	-0.59	2.91
Urban Lan. AE	0.11	*0.09*	-0.72	3.02
Human CE	0.11	*0.12*	-1.44	6.63
Animal CE	0.15	***0.01***	-1.72	6.96
Natural Eco. CE	0.10	*0.14*	-1.23	5.37
Urban Lan. CE	0.08	*0.37*	-0.85	4.26
Score Env. Attitudes	0.07	*0.63*	-0.08	2.38
Human AE nor*.	0.08	*0.37*	0.51	3.02
Animal AE nor*.	0.08	*0.41*	0.57	2.61
Animal CE nor*.	0.06	*0.69*	-0.16	2.99

Asymptotic one-sample Kolmogorov-Smirnov test. Alternative hypothesis: two-sided.

*Normalized data, using the following square root transformation:
$VAR_{norm} = \sqrt{\max(VAR + 1) - VAR}$, with *VAR* the vector of data for one image category.
