# Supplementary material for: Nature can suffer, too: behavioral evidence of empathy with ecosystems and its link to pro-environmental attitudes
Source: PeerJ. 2026 Jun 26;14:e21383. doi: 10.7717/peerj.21383 (PMC13312967; doi:10.7717/peerj.21383)
Supplement: Supplemental Information 14 — AE indicates participants’ values of behavioral affective empathy and CE, values of behavioral cognitive empathy. Significant results are shown in bold. [file peerj-14-21383-s014.pdf]

Table S8. Results of the 2 (empathy type: cognitive, affective) × 4 (image category) repeated-measures ANOVA. AE indicates participants' values of behavioral affective empathy and CE, values of behavioral cognitive empathy. Significant results are shown in bold.

**Categories**	**AE (df = 121)**				
	**Means**	**Estimate**	**SE**	**t**	***p***
human-animal	89.8-90.8	-1.01	0.78	-1.03	*0.235*
(human-natural ecosystem)	89.8-75.3	14.42	1.23	11.77	***<0.001***
(human-urban ecosystem)	89.8-74.5	15.27	1.15	12.82	***<0.001***
(animal-natural ecosystem)	90.8-75.3	15.43	1.15	13.45	***<0.001***
(animal-urban ecosystem)	90.8-74.5	16.29	1.16	13.87	***<0.001***
natural ecosystem - urban ecosystem	75.3-74.5	0.85	0.98	0.88	*0.384*

**Categories**	**CE (df = 121)**				
	**Means**	**Estimate**	**SE**	**t**	***p***
human-animal	89.8-90.8	-2.34	1.03	-2.27	***0.025***
(human-natural ecosystem)	89.8-75.3	7.37	1.23	6.00	***<0.001***
(human-urban ecosystem)	89.8-74.5	10.42	1.07	9.7	***<0.001***
(animal-natural ecosystem)	90.8-75.3	9.71	1.08	9.00	***<0.001***
(animal-urban ecosystem)	90.8-74.5	12.73	1.16	11.02	***<0.001***
natural ecosystem - urban ecosystem	75.3-74.5	3.05	0.91	3.64	***0.001***
