# Supplementary material for: Nature can suffer, too: behavioral evidence of empathy with ecosystems and its link to pro-environmental attitudes
Source: PeerJ. 2026 Jun 26;14:e21383. doi: 10.7717/peerj.21383 (PMC13312967; doi:10.7717/peerj.21383)
Supplement: Supplemental Information 15 — Spearman’s rho correlation coefficients. Significant results appear in black and non-significant results in light grey. Numerical p-values are written below each Spearman’s correlation coefficient. AE stands for Affective Empathy and CE for Cognitive Empathy. Trait empathy with humans corresponds to the ACME scale (Table S3) and trait empathy with nature corresponds to the DEN scale (Table S4). Values that were not significant after correction for multiple comparison appear in orange. [file peerj-14-21383-s015.pdf]

Table S9. Correlations between quantitative measures of state and trait empathy. Spearman's rho correlation coefficients. Significant results appear in black and non-significant results in light grey. Numerical p-values are written below each Spearman's correlation coefficient. AE stands for Affective Empathy and CE for Cognitive Empathy. Trait empathy with humans corresponds to the ACME scale (Tab. S3) and trait empathy with nature corresponds to the DEN scale (Tab. S4). All values retained the same significance after correction for multiple comparison.

	Animals - AE	*Natural Ecosystems - AE*	*Urban Ecosystems - AE*	*Humans - CE*	*Animals - CE*	*Natural Ecosystems - CE*	*Urban Ecosystems - CE*	*Trait Empathy with Humans*	*Trait Empathy with Nature*
Humans - AE	0.530 *(<.001)*	0.473 *(<.001)*	0.530 *(<.001)*	0.664 *(<.001)*	0.435 *(<.001)*	0.467 *(<.001)*	0.468 *(<.001)*	0.290 *(.001)*	0.221 *(.015)*
Animals - AE		0.553 *(<.001)*	0.562 *(<.001)*	0.488 *(<.001)*	0.652 *(<.001)*	0.579 *(<.001)*	0.476 *(<.001)*	0.259 *(.004)*	0.379 *(<.001)*
Natural Ecosystems - AE			0.721 *(<.001)*	0.360 *(<.001)*	0.394 *(<.001)*	0.558 *(<.001)*	0.507 *(<.001)*	0.102 *(.268)*	0.211 *(.021)*
Urban Ecosystems - AE				0.529 *(<.001)*	0.483 *(<.001)*	0.590 *(<.001)*	0.650 *(<.001)*	0.190 *(.038)*	0.256 *(.005)*
Humans - CE					0.692 *(<.001)*	0.659 *(<.001)*	0.755 *(<.001)*	0.359 *(<.001)*	0.218 *(.017)*
Animals - CE						0.732 *(<.001)*	0.700 *(<.001)*	0.238 *(.009)*	0.289 *(.001)*
Natural Ecosystems - CE							0.799 *(<.001)*	0.357 *(<.001)*	0.322 *(<.001)*
Urban Ecosystems - CE								0.323 *(<.001)*	0.198 *(.030)*
Trait Empathy with Humans									0.340 *(<.001)*

Computed correlation used spearman-method with pairwise-deletion.
