# Supplementary material for: Nature can suffer, too: behavioral evidence of empathy with ecosystems and its link to pro-environmental attitudes
Source: PeerJ. 2026 Jun 26;14:e21383. doi: 10.7717/peerj.21383 (PMC13312967; doi:10.7717/peerj.21383)
Supplement: Supplemental Information 16 — Spearman’s rho correlation coefficients. Significant results appear in black and non-significant results in light grey. Numerical p-values are written below each Spearman’s correlation coefficient. AE stands for Affective Empathy and CE for Cognitive Empathy. Trait empathy with humans corresponds to the ACME scale (Table S3) and trait empathy with nature corresponds to the DEN scale (Table S4). Pro-environmental attitudes correspond to the EAI scale (Table S5). No change in significance was observed after correction for multiple comparison. [file peerj-14-21383-s016.pdf]

Table S10. Correlations of state and trait empathy measures with pro-environmental attitudes. Spearman's rho correlation coefficients. Significant results appear in black and non-significant results in light grey. Numerical p-values are written below each Spearman's correlation coefficient. AE stands for Affective Empathy and CE for Cognitive Empathy. Trait empathy with humans corresponds to the ACME scale (Tab. S3) and trait empathy with nature corresponds to the DEN scale (Tab. S4). Pro-environmental attitudes correspond to the EAI scale (Tab. S5). All values retained the same significance after correction for multiple comparison.

	Pro-environmental attitudes
Humans - AE	0.014<br>*(.877)*
Animals - AE	0.268<br>*(.003)*
Natural Ecosystems - AE	0.162<br>*(.075)*
Urban Ecosystems - AE	0.064<br>*(.484)*
Humans - CE	0.031<br>*(.734)*
Animals - CE	0.258<br>*(.004)*
Natural Ecosystems - CE	0.218<br>*(.016)*
Urban Ecosystems - CE	0.159<br>*(.081)*
Trait Empathy with Humans	0.209<br>*(.023)*
Trait Empathy with Nature	0.337<br>*(<.001)*

Computed correlation used Spearman-method with pairwise-deletion.
