# Supplementary material for: Nature can suffer, too: behavioral evidence of empathy with ecosystems and its link to pro-environmental attitudes
Source: PeerJ. 2026 Jun 26;14:e21383. doi: 10.7717/peerj.21383 (PMC13312967; doi:10.7717/peerj.21383)
Supplement: Supplemental Information 18 — Models were obtained using the lm() function of the R language, aimed to fit linear models to datasets. AE stands for Affective Empathy, CE for Cognitive Empathy and HNC for Human-Nature Connectedness. [file peerj-14-21383-s018.pdf]

Table S12. Effects of demographical variables on behavioral empathy for the human's pictures category. Models were obtained using the lm() function of the R language, aimed to fit linear models to datasets. AE stands for Affective Empathy, CE for Cognitive Empathy and HNC for Human-Nature Connectedness. Significant results are shown in bold. Results with p-values <0.1 are shown in light gray and bold.

	Model 1: Humans AE			***Model 2: Humans CE***		
Predictors	**Estimates**	**CI**	***p***	**Estimates**	**CI**	***p***
(Intercept)	87.41	80.99 – 93.83	***<0.001***	83.67	71.92 – 95.42	***<0.001***
Sex [Other]	13.98	-3.80 – 31.77	*0.122*	-0.70	33.27 – 31.88	*0.966*
Sex [Female]	5.20	1.69 – 8.71	***0.004***	6.52	0.09 – 12.95	***0.047***
HNC	1.17	-0.45 – 2.80	*0.155*	2.48	-0.50 – 5.45	*0.102*
Age	-1.23	-2.88 – 0.42	*0.143*	-1.18	-4.20 – 1.85	*0.442*
Pet During Childhood	-0.51	-5.78 – 4.76	*0.848*	-4.80	-14.45 – 4.86	*0.327*
Pet Since Adulthood	-3.16	-10.74 – 4.42	*0.410*	-2.33	-16.21 – 11.55	*0.740*
Pet Forever	-2.83	-7.90 – 2.24	*0.272*	-6.10	-15.40 – 3.19	*0.196*
Semi-rural Origin	3.20	-1.16 – 7.56	*0.149*	-2.33	-10.31 – 5.66	*0.565*
Urban Origin	0.48	-4.28 – 5.24	*0.841*	-3.69	-12.41 – 5.03	*0.403*
City-center Origin	-2.94	-8.13 – 2.25	*0.264*	-10.32	-19.83 – -0.81	***0.034***
Observations	122			122		
R^2 / R^2 adjusted	0.081 / -0.001			0.157 / 0.081		
