# Supplementary material for: Nature can suffer, too: behavioral evidence of empathy with ecosystems and its link to pro-environmental attitudes
Source: PeerJ. 2026 Jun 26;14:e21383. doi: 10.7717/peerj.21383 (PMC13312967; doi:10.7717/peerj.21383)
Supplement: Supplemental Information 19 — Models were obtained using the lm() function of the R language, aimed to fit linear models to datasets. Note: due to the normalization applied to these variables, negative estimates represent a positive effect of the variables. AE stands for Affective Empathy, CE for Cognitive Empathy and HNC for Human-Nature Connectedness. [file peerj-14-21383-s019.pdf]

Table S13. Effects of demographical variables on behavioral empathy for the animal's pictures category. Models were obtained using the lm() function of the R language, aimed to fit linear models to datasets. Note: due to the normalization applied to these variables, negative estimates represent a positive effect of the variables. AE stands for Affective Empathy, CE for Cognitive Empathy and HNC for Human-Nature Connectedness. Significant results are shown in bold. Results with p-values <0.1 are shown in light gray and bold.

	Model 1: Animals AE			***Model 2: Animals CE***		
Predictors	**Estimates**	**CI**	***p***	**Estimates**	**CI**	***p***
(Intercept)	3.17	2.19 – 4.14	***<0.001***	4.17	2.82 – 5.53	***<0.001***
Sex [Other]	-2.10	-4.81 – 0.60	*0.126*	-1.48	-5.23 – 2.27	*0.436*
Sex [Female]	-0.56	-1.10 – -0.03	***0.040***	-0.83	-1.57 – -0.09	***0.028***
HNC	0.07	-0.18 – 0.32	*0.580*	-0.14	-0.49 – 0.20	*0.412*
Age	0.15	-0.10 – 0.40	*0.234*	0.14	-0.21 – 0.49	*0.426*
Pet During Childhood	-0.23	-1.03 – 0.57	*0.569*	-0.45	-1.56 – 0.66	*0.422*
Pet Since Adulthood	-0.25	-1.40 – 0.90	*0.669*	0.00	-1.59 – 1.60	*0.995*
Pet Forever	0.08	-0.69 – 0.85	*0.832*	0.12	-0.95 – 1.19	*0.822*
Semi-rural Origin	-0.20	-0.86 – 0.46	*0.551*	0.24	-0.68 – 1.15	*0.613*
Urban Origin	0.49	-0.23 – 1.22	*0.180*	0.46	-0.54 – 1.46	*0.365*
City-center Origin	0.52	-0.27 – 1.31	*0.192*	1.05	-0.05 – 2.14	***0.060***
Observations	122			122		
R^2 / R^2 adjusted	0.117 / 0.037			0.106 / 0.026		
