# Supplementary material for: Nature can suffer, too: behavioral evidence of empathy with ecosystems and its link to pro-environmental attitudes
Source: PeerJ. 2026 Jun 26;14:e21383. doi: 10.7717/peerj.21383 (PMC13312967; doi:10.7717/peerj.21383)
Supplement: Supplemental Information 20 — Models were obtained using the lm() function of the R language, aimed to fit linear models to datasets. AE stands for Affective Empathy, CE for Cognitive Empathy and HNC for Human-Nature Connectedness. [file peerj-14-21383-s020.pdf]

**Table S14. Effects of demographical variables on behavioral empathy for the natural ecosystem's picture category.** Models were obtained using the lm() function of the R language, aimed to fit linear models to datasets. AE stands for Affective Empathy, CE for Cognitive Empathy and HNC for Human-Nature Connectedness. Significant results are shown in bold. Results with p-values <0.1 are shown in light gray and bold.

| | ***Model 1: Natural Eco. AE*** | | | ***Model 2: Natural Eco. CE*** | | |
|---|---|---|---|---|---|---|
| ***Predictors*** | **Estimates** | **CI** | ***p*** | **Estimates** | **CI** | ***p*** |
| *(Intercept)* | 78.28 | 67.20 – 89.35 | ***<0.001*** | 74.03 | 61.07 – 87.00 | ***<0.001*** |
| *Sex [Other]* | 17.51 | -13.20 – 48.21 | *0.261* | 9.17 | -26.77 – 45.12 | *0.614* |
| *Sex [Female]* | 2.43 | -3.63 – 8.49 | *0.429* | 9.44 | 2.35 – 16.53 | ***0.010*** |
| *HNC* | -0.95 | -3.76 – 1.85 | *0.502* | 2.14 | -1.15 – 5.42 | *0.200* |
| *Age* | -1.71 | -4.56 – 1.14 | *0.236* | -1.41 | -4.75 – 1.93 | *0.404* |
| *Pet During Childhood* | -3.09 | -12.19 – 6.01 | *0.502* | -0.82 | -11.47 – 9.84 | *0.879* |
| *Pet Since Adulthood* | -10.62 | -23.70 – 2.46 | *0.111* | -3.97 | -19.28 – 11.35 | *0.609* |
| *Pet Forever* | -2.47 | -11.23 – 6.28 | *0.577* | -6.72 | -16.97 – 3.53 | *0.197* |
| *Semi-rural Origin* | -1.55 | -9.08 – 5.97 | *0.683* | -4.66 | -13.46 – 4.15 | *0.297* |
| *Urban Origin* | -1.98 | -10.20 – 6.23 | *0.633* | -1.19 | -10.81 – 8.43 | *0.807* |
| *City-center Origin* | -4.61 | -13.57 – 4.35 | *0.310* | -12.77 | -23.26 – -2.27 | ***0.018*** |
| *Observations* | 122 | | | 122 | | |
| $R^2$ / $R^2$ *adjusted* | 0.081 / -0.001 | | | 0.157 / 0.081 | | |
