# Supplementary material for: Nature can suffer, too: behavioral evidence of empathy with ecosystems and its link to pro-environmental attitudes
Source: PeerJ. 2026 Jun 26;14:e21383. doi: 10.7717/peerj.21383 (PMC13312967; doi:10.7717/peerj.21383)
Supplement: Supplemental Information 21 — Models were obtained using the lm() function of the R language, aimed to fit linear models to datasets. AE stands for Affective Empathy, CE for Cognitive Empathy and HNC for Human-Nature Connectedness. [file peerj-14-21383-s021.pdf]

Table S15. Effects of demographical variables on behavioral empathy for the urban ecosystem's pictures category. Models were obtained using the lm() function of the R language, aimed to fit linear models to datasets. AE stands for Affective Empathy, CE for Cognitive Empathy and HNC for Human-Nature Connectedness. Significant results are shown in bold. Results with p-values <0.1 are shown in light gray and bold.

	Model 1: Urban Eco. AE			***Model 2: Urban Eco. CE***		
Predictors	**Estimates**	**CI**	***p***	**Estimates**	**CI**	***p***
(Intercept)	75.16	63.89 – 86.43	***<0.001***	76.02	62.82 – 89.22	***<0.001***
Sex [Other]	21.80	-9.44 – 53.04	*0.170*	-13.75	-50.35 – 22.84	*0.458*
Sex [Female]	6.10	-0.06 – 12.27	***0.052***	7.80	0.57 – 15.02	***0.035***
HNC	-1.30	-4.15 – 1.56	*0.369*	1.25	-2.09 – 4.60	*0.460*
Age	-1.34	-4.24 – 1.56	*0.361*	-0.10	-3.49 – 3.30	*0.954*
Pet During Childhood	-1.29	-10.55 – 7.97	*0.782*	-4.12	-14.97 – 6.72	*0.453*
Pet Since Adulthood	-3.95	-17.27 – 9.36	*0.557*	-6.94	-22.54 – 8.65	*0.380*
Pet Forever	-5.98	-14.89 – 2.93	*0.186*	-8.96	-19.40 – 1.48	***0.092***
Semi-rural Origin	0.46	-7.20 – 8.11	*0.906*	-6.50	-15.47 – 2.47	*0.154*
Urban Origin	0.17	-8.19 – 8.54	*0.967*	-5.05	-14.84 – 4.75	*0.310*
City-center Origin	-7.94	-17.06 – 1.18	***0.087***	-12.30	-22.99 – -1.62	***0.024***
Observations	122			122		
R^2 / R^2 adjusted	0.114 / 0.034			0.115 / 0.035		
